# Cisplatin-induced cell death increases the degradation of the MRE11-RAD50-NBS1 complex through the autophagy/lysosomal pathway

**DOI:** 10.1038/s41418-022-01100-1

**Published:** 2022-12-08

**Authors:** Alejandro Belmonte-Fernández, Joaquín Herrero-Ruíz, María Galindo-Moreno, M. Cristina Limón-Mortés, Mar Mora-Santos, Carmen Sáez, Miguel Á. Japón, Maria Tortolero, Francisco Romero

**Affiliations:** 1 Departamento de Microbiología, Facultad de Biología, Universidad de Sevilla, Sevilla, E-41012 Spain; 2 Instituto de Biomedicina de Sevilla (IBiS) and Departamento de Anatomía Patológica, Hospital Universitario Virgen del Rocío, Sevilla, E-41013 Spain

**Keywords:** Ubiquitin ligases, Macroautophagy, Cancer

## Abstract

Cisplatin and other platinum-based anticancer agents are among the most widely used chemotherapy drugs in the treatment of different types of cancer. However, it is common to find patients who respond well to treatment at first but later relapse due to the appearance of resistance to cisplatin. Among the mechanisms responsible for this phenomenon is the increase in DNA damage repair. Here, we elucidate the effect of cisplatin on the MRN (MRE11-RAD50-NBS1) DNA damage sensor complex. We found that the tumor suppressor FBXW7 is a key factor in controlling the turnover of the MRN complex by inducing its degradation through lysosomes. Inhibition of lysosomal enzymes allowed the detection of the association of FBXW7-dependent ubiquitylated MRN with LC3 and the autophagy adaptor p62/SQSTM1 and the localization of MRN in lysosomes. Furthermore, cisplatin-induced cell death increased MRN degradation, suggesting that this complex is one of the targets that favor cell death. These findings open the possibility of using the induction of the degradation of the MRN complex after genotoxic damage as a potential therapeutic strategy to eliminate tumor cells.

## Introduction

Cisplatin (CDDP, *cis*-diamminedichloroplatinum II) is a key drug in the fight against cancer. Since its discovery, it has been widely used in chemotherapy treatments. Currently, platinum-based drugs are used for the treatment of many cancers, including cervical, ovarian, testicular, head and neck, breast, bladder, esophageal, stomach, prostate, and lung cancers [1]. Also, to treat Hodgkin and non-Hodgkin lymphomas, neuroblastoma, sarcomas, multiple myeloma, melanoma, and mesothelioma [2]. Cisplatin, activated by intracellular hydrolysis of chlorides, binds to DNA to form monoadducts as well as intra- and interstrand DNA cross-links [3]. As a result, it causes inhibition of replication and transcription, arrests the cell cycle, and, since cells cannot repair DNA damage, induces cell death [4]. However, the use of cisplatin is often limited by inherent or acquired chemoresistance, leading to therapeutic failure. This is especially relevant after repeated cycles of therapy, which can enhance the ability to repair DNA damage to avoid cell death. Various mechanisms are involved in the repair of DNA damage caused by cisplatin, such as nucleotide excision repair, homologous recombination (HR), and nonhomologous end joining (NHEJ), among others [5]. These observations are the basis for the use of repair inhibitors to enhance the therapeutic effects of DNA-damaging drugs [6].

Double-stranded breaks (DSBs) can be recognized by either the MRN (MRE11-RAD50-NBS1) or the KU70/KU80 complexes. Binding of the first complex prepares the breaks for HR repair, while binding of the second leads to NHEJ [7]. The MRN complex is made up of two subunits of MRE11, two subunits of RAD50, and two subunits of NBS1, and functions in both DSB detection and repair [8]. The recruitment of MRN to DNA damage sites is mediated by its association with several proteins, such as γH2AX or RAD17 [9, 10]. MRE11 is the key component of the MRN complex, exhibiting both endonuclease and exonuclease activities from 3′-5′ [11]. The proteins UBQLN4, C1QBP, p97/VCP or GRB2 regulate the nuclease activity of MRE11 by preventing binding of MRE11 to DNA or by removing MRE11 from damaged chromatin [12–15].

It has been described that the decrease in DYNLL1, which binds to and inhibits the resection activity of MRE11, restored HR in *BRCA1* mutant cells inducing resistance to cisplatin [16]. In fact, *BRCA1* mutant patients with low *DYNLL1* expression significantly correlated with poor progression-free survival. Furthermore, it has also been shown that *NBS1* or *RAD50* disruption enhanced cisplatin chemosensitization in human tumor cells [17, 18]. These and other findings relate the activity of the MRN complex to platinum-based chemoresistance.

In yeast, activation of the DNA damage checkpoint induced the degradation of core histones and the recruitment of ubiquitin ligases and the proteasome to chromatin [19]. The two main protein degradation pathways in eukaryotic cells are the ubiquitin-dependent proteasome system and the autophagy/lysosomal pathway. In both cases, ubiquitylated proteins are recognized and degraded by the proteasome or lysosome, respectively [20]. Three enzymes are involved in the ubiquitylation of proteins: E1 ubiquitin activating enzyme, E2 ubiquitin conjugating enzyme, and E3 ubiquitin protein ligase. The E3 ubiquitin ligases are responsible for substrate recognition and thus determine the specificity of the process. The proteasome recognizes ubiquitylated substrates through its ubiquitin receptors, removes ubiquitins, and proteins are digested to peptides [21]. Alternatively, p62/SQSTM1 and other autophagy adaptor proteins target ubiquitylated proteins to the entry of autophagosomes through the LC3-II protein. Autophagosomes fuse to the lysosome to degrade content, including p62 and ubiquitylated proteins [22].

SCF (SKP1-CUL1-F-box protein) is a multisubunit ubiquitin ligase comprised of three invariant components and an interchangeable component, the F-box protein, which specifically binds to substrates. FBXW7, one of the F-box proteins of the SCF complex, is encoded by a gene that appears frequently mutated or suppressed in human cancers. For this and other reasons, FBXW7 is considered a tumor suppressor, although it may depend on the cellular context [23, 24].

Based on a previous proteomic study, this paper analyzes the association of FBXW7 with the MRN complex and its role in response to platinum-based chemotherapies. We discuss the importance of promoting MRN degradation by SCF (FBXW7) to avoid platinum resistance.

## Results

### FBXW7 induces lysosomal degradation of the MRN complex

**Fig. 1:**
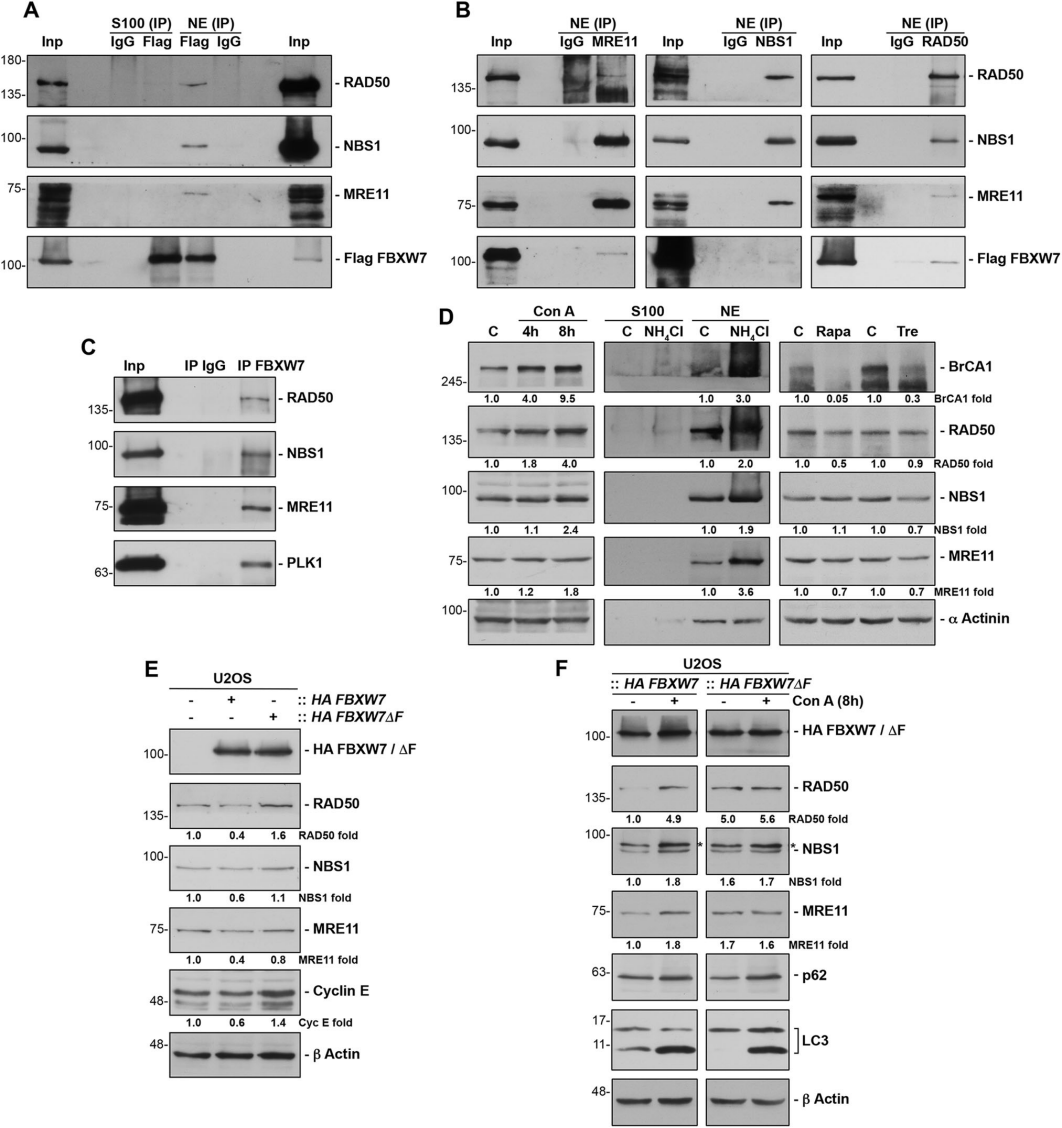
FBXW7 interacts with the MRN complex and induces its degradation by lysosomes. **A** COS-7 cells were transiently transfected with pCDNA3 Flag FBXW7 and nuclear (NE) and cytosolic extracts (S100) immunoprecipitated with anti-Flag or normal mouse serum (IgG) as a control. Immunoprecipitated materials were analyzed by Western blotting. Inp: the input lane was loaded with 1/20 of the extract used in each assay. Data are representative of two independent experiments. **B** Similar to A, but using anti-MRE11, anti-NBS1, or anti-RAD50 antibodies. Data are representative of two independent experiments. **C** Nuclear extracts of U2OS cells were used to immunoprecipitate endogenous FBXW7, and the obtained complexes were analyzed by immunoblotting. IgG: immunoprecipitation using normal rabbit serum, as a control. Inp: the input lane was loaded with 1/20 of the nuclear extract. Western blot against PLK1 was used as a control of the efficiency of FBXW7 immunoprecipitation. Data are representative of three independent experiments. **D** U2OS cells were treated with Con A (for the indicated times), NH_4_Cl (24 h), rapamycin (Rapa, 16 h) or trehalose (Tre, 24 h), whole cell extracts or nuclear and cytosolic extracts (where indicated) prepared, and membranes immunoblotted with the indicated antibodies. C: extracts of untreated cells. Western blots are representative of at least three replicates. **E** Whole cell extracts from U2OS or transduced U2OS::*HA FBXW7* or U2OS::*HA FBXW7ΔF* cells were electroblotted and probed with the indicated antibodies. Western blot against cyclin E was used as a control of the efficiency of *FBXW7* or *FBXW7ΔF* expression. **F** U2OS::*HA FBXW7* and U2OS::*HA FBXW7ΔF* cells were treated or not with Con A (8 h) and analyzed by immunoblotting. *non-specific band from a previous immunoblotting. Data from E and F are representative results from three independent experiments. Quantitative fold change in proteins was determined relative to the loading control (α actinin or β actin).

In previous mass spectrometry studies of Flag FBXW7 immunoprecipitates from transiently transfected COS-7 cells [25], we identified a single peptide corresponding to MRE11 residues 468-478 (Supplementary Fig. S1A). To verify that FBXW7 and Mre11 are associated in vivo in normally growing cells, the presence of MRE11 in Flag FBXW7 immune complexes was analyzed by Western blotting. Figure 1A shows that not only endogenous MRE11, but also NBS1 and RAD50 interact directly or indirectly with Flag FBXW7. It also shows that these interactions are specifically detected in the nuclear fraction of cells. Furthermore, reciprocal co-immunoprecipitation experiments revealed that Flag FBXW7 also coprecipitated with MRE11, NBS1 and RAD50 immune complexes, respectively (Fig. 1B). But, most importantly, FBXW7 and the MRN complex at endogenous levels bind to each other under unstimulated physiological conditions, as we were able to demonstrate using an anti-FBXW7 antibody (Fig. 1C and Supplementary Fig. S1B). As at least in our hands, commercial FBXW7 antibodies do not work well in Western blot analyses, we used coimmunoprecipitation of PLK1 as a positive control for the good functioning of FBXW7 immunoprecipitation [25]. To determine whether the SCF (FBXW7) ubiquitin ligase could be involved in the stability of the MRN complex proteins, we first analyzed the effect of proteasomal or lysosomal inhibitors on the amount of MRN complex components. Treatment of U2OS cells with the proteasome inhibitor MG132 did not increase the levels of MRE11, NBS1 or RAD50 (Supplementary Fig. S1C and Supplementary Table S1). However, inhibition of lysosomal activity by concanamycin A or ammonium chloride did induce an increase in the amount of MRN in the nuclear fraction of these cells (left and center panels of Fig. 1D, respectively, and Supplementary Fig. S1D). The accumulation of BrCA1 after ammonium chloride treatment was used as a control [26]. In addition, activation of the autophagy/lysosome pathway using rapamycin or trehalose caused a slight decrease in the levels of the proteins of the MRN complex (right panels of Fig. 1D and Supplementary Fig. S1D). Therefore, MRN complex proteins are degraded through the lysosome. Next, we constructed lentivirus expressing *FBXW7* or *FBXW7ΔF*, which encodes a dominant negative form of FBXW7 [27], and infected U2OS cells to study their effects on the MRN complex. We found that FBXW7 decreased the amount of the three proteins of the complex, while FBXW7ΔF prevented this reduction (Fig. 1E and Supplementary Fig. S1E). Cyclin E was used as a known protein that is degraded by FBXW7 [28]. Furthermore, the treatment with concanamycin A avoided the degradation of MRN due to SCF (FBXW7) (Fig. 1F and Supplementary Fig. S1F). Together, these results indicate that SCF (FBXW7) induces the degradation of the MRN complex by the lysosome.

### MRN degradation is mediated by the Ub-p62-LC3 pathway

**Fig. 2:**
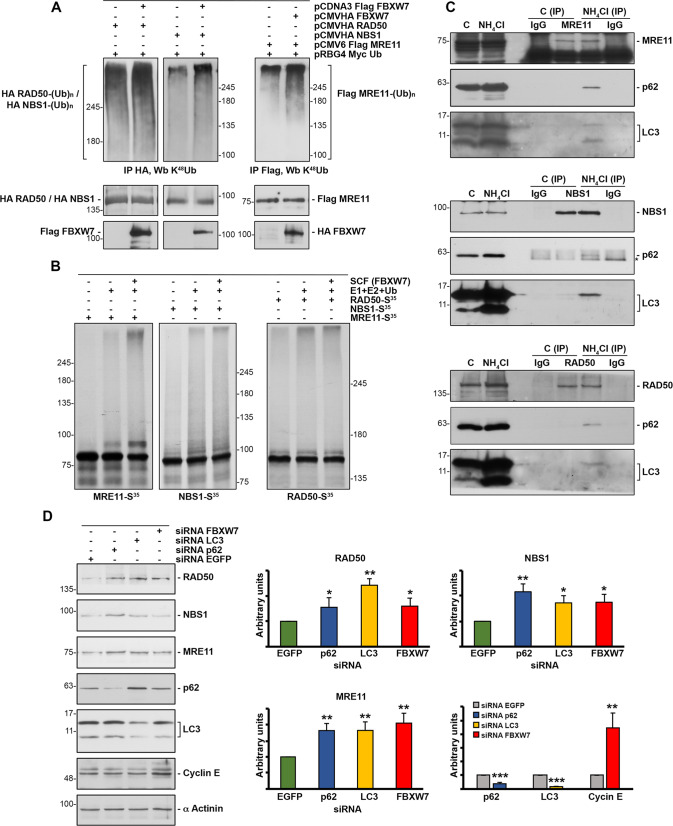
SCF (FBXW7) polyubiquitylates MRN proteins and these associate with p62/LC3. **A** HEK293T cells were transfected with the indicated plasmids and treated with ammonium chloride 4 h before collection. Extracts were prepared and polyubiquitylated Flag MRE11, HA NBS1, or HA RAD50 visualized after Western blot analysis of the corresponding immunoprecipitations. Brackets mark a ladder of bands corresponding to polyubiquitylated proteins. **B** In vitro ubiquitylation assay of in vitro transcribed and translated MRE11, NBS1 or RAD50 labeled with [^35^S] was carried out in the presence or absence of the following products: recombinant SCF (FBXW7) complex expressed in Sf21 insect cells, E1 (His_6_-E1), E2 (His_6_-UbcH3 and UbcH5a), and ubiquitin (Ub). The samples were incubated at 30 °C for 1 h and analyzed by SDS-PAGE and autoradiography. Polyubiquitylated proteins appear as a ladder of bands. **C** U2OS cells were treated or not with ammonium chloride for 24 hours prior to harvesting. NP40 extracts were used to immunoprecipitate endogenous MRE11, NBS1, or RAD50. Normal mouse or rabbit sera (IgG) were used as a control. Complexes were analyzed by immunoblotting. C: extracts from U2OS cells; NH_4_Cl: extracts from U2OS cells treated with ammonium chloride. *non-specific band. **D** U2OS cells were interfered with the indicated siRNAs, and whole cell extracts were blotted with the designated antibodies. The graphs show the quantification of protein levels using ImageJ software. Error bars represent the SD (n = 3). *p < 0.05, **p < 0.01, ***p < 0.001 (Student’s *t*-test).

To learn more about the degradation of the MRN complex by SCF (FBXW7)/lysosome, we analyzed the ability of SCF (FBXW7) to target proteins from the MRN complex for ubiquitylation in vivo. HEK293T cells expressing epitope-tagged MRE11, NBS1 or RAD50, and Myc Ub in the presence or absence of epitope-tagged FBXW7 were used to assess MRN ubiquitylation. We observed an increase in K48-linked polyubiquitylation in each of the proteins when cells were also transfected with *FBXW7* (Fig. 2A). However, when we examined the in vitro ubiquitylation of transcribed and translated [^35^S]-labeled MRE11, NBS1 or RAD50 by recombinant SCF (FBXW7) produced in insect cells, only high molecular weight ubiquitylated forms of MRE11 were detected (Fig. 2B). These results suggest that MRE11 is the direct substrate of FBXW7 and that, once bound, SCF (FBXW7) also targets NBS1 and RAD50 for ubiquitylation.

Degradation of ubiquitylated proteins through the autophagy/lysosome pathway is usually mediated by an ubiquitin-binding protein (such as p62), which binds directly to ubiquitylated proteins and to autophagosome-associated LC3 [29]. To determine the implication of these proteins in MRN degradation, we explored the potential in vivo association of the MRN complex with p62/LC3 by means of coimmunoprecipitation assays. We detected the association between MRN and p62/LC3 by inhibiting lysosomal enzymes with ammonium chloride, an interaction that was not observed using extracts from untreated cells, probably because proteins were degraded by the lysosome (Fig. 2C). Even more, knockdown experiments showed that reduced expression of *p62* or *LC3* increased the levels of the MRN complex proteins (Fig. 2D). Similar results were obtained using cells transfected with siRNA FBXW7 or an *FBXW7*^-/-^ cell line (Fig. 2D and Supplementary Fig. S2). Overall, these data demonstrate that degradation of MRN by the lysosome is mediated by p62/LC3.

### SCF (FBXW7) promotes the association of MRN with p62/LC3 in the nuclei of U2OS cells

**Fig. 3:**
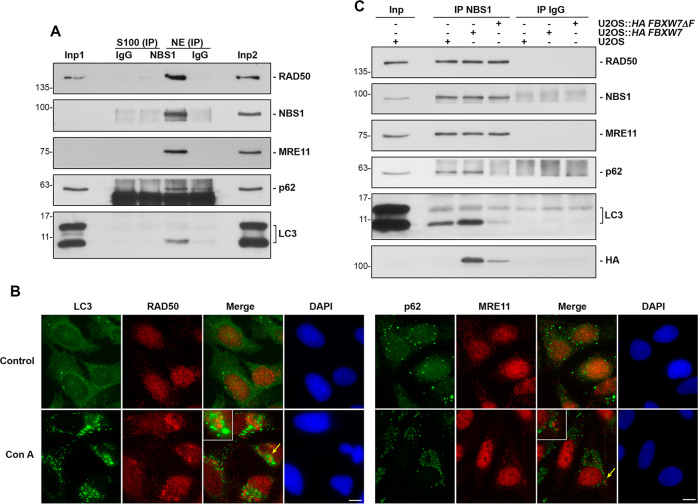
The interaction of MRN with p62/LC3 occurs in the nuclei of U2OS cells and is promoted by FBXW7. **A** Cytosolic (S100) and nuclear (NE) extracts were prepared from U2OS cells treated with ammonium chloride. Immunoprecipitations with an anti-NBS1 rabbit monoclonal antibody or with normal rabbit serum were performed and complexes immunoblotted with the indicated antibodies. Inp1, 2: the input lanes were loaded with 1/20 of the extract used in each assay (Inp1: S100, Inp2: NE). **B** U2OS cells treated or not with Con A for 8 h were analyzed by light microscopy. Yellow arrows indicate magnified areas (x2). The bars represent 10 μm. **C** Similar to A, but using only nuclear extracts from the indicated cell lines treated with ammonium chloride.

The proteins of the MRN complex are mainly located in the nucleus [30]. However, p62 and LC3 are found in both the nucleus and the cytoplasm [29]. To find out where the association of MRN with p62/LC3 takes place, we performed subcellular fractionation experiments. By blocking lysosomal activity with ammonium chloride, we showed that the in vivo association of MRN with p62/LC3 occurs exclusively in the nuclear fraction of U2OS cells (Fig. 3A). However, these assays do not rule out that there are also associations in the cytoplasm, mainly because of the detection limitations of co-immunoprecipitation experiments. Although, they would clearly be less relevant than those in the nucleus. In the same vein, light microscopy examination revealed that endogenous RAD50 (or MRE11) and LC3 (or p62) are localized in the periphery of the nuclei of cells treated with concanamycin A, which is not observed in untreated cells (Fig. 3B and Supplementary Fig. S3A, B, C). Therefore, we can conclude that the MRN-p62/LC3 interaction occurs primarily in the nuclei of U2OS cells.

To establish the involvement of SCF (FBXW7) in the MRN-p62/LC3 association, we used U2OS cells expressing *FBXW7* or *FBXW7ΔF* from lentiviral constructs. We found that FBXW7 increased the MRN-p62/LC3 association. But, most importantly, the dominant negative mutant of FBXW7 almost completely prevented the interaction (Fig. 3C and Supplementary Fig. S3D). These results indicate that SCF (FBXW7) is responsible for the MRN-p62/LC3 association in vivo.

### FBXW7 induces the lysosomal localization of the MRN complex

**Fig. 4:**
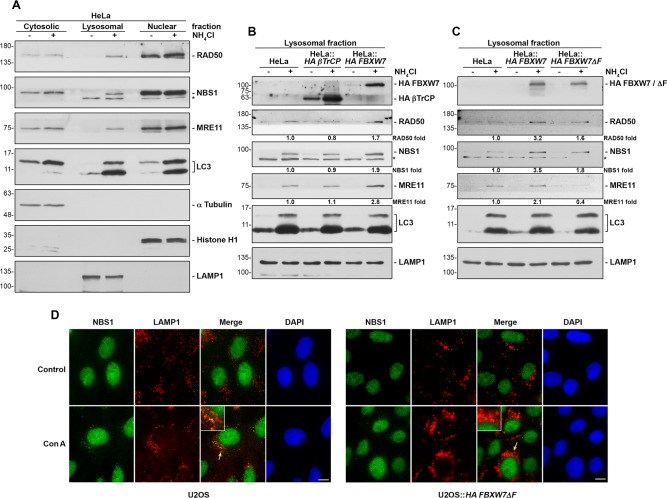
The MRN complex is localized in lysosomes in a FBXW7-dependent manner. **A** Subcellular fractions were prepared from HeLa cells treated or not with ammonium chloride for 24 h, as described in Materials and Methods. The amount of proteins used from the cytosolic, lysosomal, and nuclear fractions represented 5%, 30%, and 2.5% of the total proteins of each fraction, respectively. The extracts were immunoblotted with the indicated antibodies. The purity of the fractions was tested with anti-α Tubulin (cytosolic fraction), anti-Histone H1 (nuclear fraction), and anti-LAMP1 (lysosome-rich fraction). **B** The lysosomal fractions of HeLa, HeLa::*HA βTrCP*, and HeLa::*HA FBXW7* treated or not with ammonium chloride for 24 h were prepared as in A. Equal amounts of protein were analyzed by SDS-PAGE and immunoblot. **C**. Similar to B using the indicated cell lines. *non-specific band. Quantitative fold change in proteins was determined relative to the LC3 protein. Data from **A**, **B**, and **C** are representative results from 1-3 independent experiments. **D** U2OS and U2OS::*HA FBXW7ΔF* cells treated or not with Con A for 8 h were analyzed by light microscopy. Yellow arrows indicate magnified areas (x2). The bars represent 10 μm.

As we have shown above, FBXW7 promotes, on the one hand, the association of the MRN complex with the autophagy machinery in cell nuclei and, on the other, the degradation of the complex by lysosomes. Then, we decided to carry out a more precise study to distinguish between the cytosol and the microsomal fraction enriched in lysosomes to determine whether MRN could also be localized in that fraction. We were also interested in knowing the potential role of FBXW7 in that localization. To do that, we first tested various cell lines and found that HeLa cells were the best model to perform this research. Next, we prepared cytosolic, lysosomal, and nuclear fractions and observed that inhibition of lysosomal activity with ammonium chloride allowed us to detect the components of the MRN complex in the lysosome-rich fraction (Fig. 4A and Supplementary Fig. S4A). Furthermore, the presence of FBXW7 from a lentiviral construct increased the lysosomal localization of MRN, while another F-box protein such as βTrCP did not (Fig. 4B, Supplementary Fig. S4B, and Supplementary Table S2). Even more, the dominant negative version of FBXW7 reduced the presence of MRN in lysosomes compared to FBXW7, suggesting that this localization depends on its polyubiquitylation by SCF (FBXW7) (Fig. 4C, Supplementary Fig. S4C, and Supplementary Table S2). Similarly, microscopy studies of normally growing cells showed that blocking lysosomal enzymes made it possible to detect NBS1 in lysosomes visualized with an anti-LAMP1 antibody (Fig. 4D, left panels, and Supplementary Fig. S4D). However, the expression of FBXW7ΔF avoided this localization (Fig. 4D, right panels, and Supplementary Fig. S4D). Taken together, these results suggest that the MRN-p62/LC3 association initially occurs in the nucleus, and then the MRN proteins move to lysosomes to be degraded by the enzymes localized there.

### The degradation of the MRN complex by the SCF (FBXW7)/lysosome pathway is increased after DNA damage-induced cell death

**Fig. 5:**
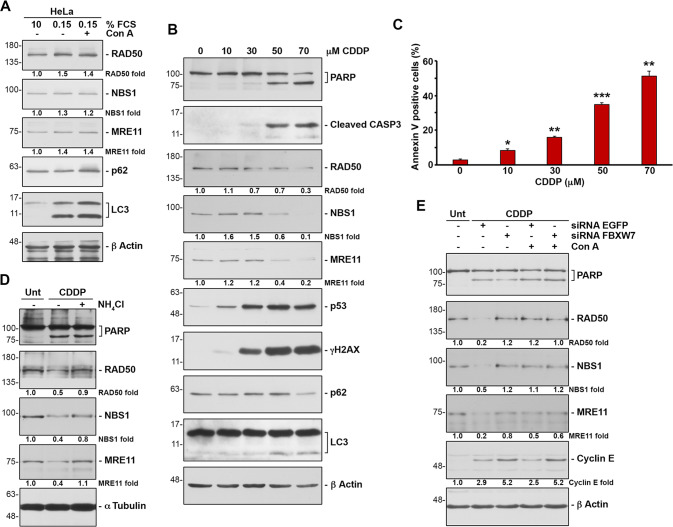
Cisplatin-induced cell death increases the degradation of the MRN complex through FBXW7/lysosome. **A** HeLa cells were grown in serum containing complete medium (10% FCS) or in serum starved medium (0.15% FCS) treated or not with Con A 8 h before harvesting. Whole cell extracts were electroblotted and probed with different antibodies. **B** U2OS cells were incubated with different concentrations of cisplatin (CDDP) for 24 h. Whole cell extracts were analyzed by immunoblotting. **C** Percentage of annexin V positive U2OS cells treated as in B detected by flow cytometry. Error bars represent the SD (*n* = 3). *p < 0.05, **p < 0.01, ***p < 0.001 (Student’s *t*-test). **D** U2OS cells were treated or not with CDDP (50  μM) or with CDDP and ammonium chloride for 24 h and whole cell extracts analyzed by Western blotting. Unt: untreated cells. **E** U2OS cells transfected with siRNA FBXW7 (or siRNA EGFP as a control) were treated with CDDP (50 μM) for 24 h. Whole cell extracts were electroblotted and probed with the indicated antibodies. All of data are representative of at least two independent experiments. Quantitative fold change in proteins was determined relative to the loading control (α tubulin or β actin).

**Fig. 6:**
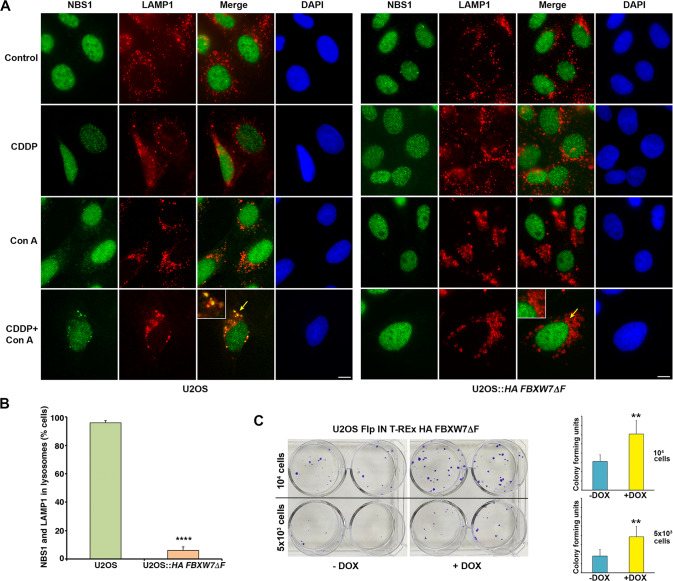
Cisplatin-induced increase of MRN in lysosomes is prevented by FBXW7ΔF. **A** U2OS and U2OS::*HA FBXW7ΔF* cells treated or not with cisplatin (CDDP) for 24 h and/or Con A for 8 h were analyzed by light microscopy. Yellow arrows indicate magnified areas (x2). The bars represent 10 μm. **B** Percentage of U2OS versus U2OS::*HA FBXW7ΔF* cells displaying NBS1 in lysosomes (visualized with anti-LAMP1) after cisplatin (CDDP) and Con A treatment. A minimum of 50 cells were analyzed in triplicate for each condition. Student’s *t*-test was used to determine statistical significance. ****p < 0.0001. **C** Colony assays showing cell viability for U2OS Flp In T-REx HA FBXW7ΔF cells induced or not with doxycycline (DOX) and treated with CDDP (30 μM) for 4 h. Colony forming units from initial 5 × 10^3^ and 10^4^ cells of each condition were quantified using ImageJ software, and represented in graphs. Error bars represent the SD (*n* = 3). **p < 0.01 (Student’s *t*-test).

Autophagy is induced by starvation and other cellular stressors such as hypoxia, DNA damage, endoplasmic reticulum stress, or pathogen infection [31]. To understand the physiological conditions that increase MRN degradation by the autophagy/lysosome pathway, we first evaluated the effect of serum deprivation. We cultured HeLa cells for 3 days in medium supplemented with 0.15% FCS and compared the amount of MRN with that found in cells growing in complete medium. We observed that starvation-induced autophagy, detected by increased levels of LC3-II and decreased levels of p62, did not induce degradation of the proteins of the MRN complex (Fig. 5A and Supplementary Table S3). As a control of autophagy induction, we blocked lysosomal enzymes with concanamycin A under the same conditions. Next, given the role of the MRN complex as a sensor of DNA damage, we explored the effect of DNA-damaging agents such as cisplatin or doxorubicin on MRN levels. We performed a dose-response assessment between the concentration of cisplatin and the amount of protein of the MRN complex after 24 hours of treatment. We found that the amount of MRN decreased when the concentration of cisplatin was sufficient to induce apoptosis, as determined by PARP cleavage, caspase 3 activation, and the appearance of cells positive for annexin V (Fig. 5B, C, and Supplementary Table S3). Similar results were obtained when cells were treated with doxorubicin (Supplementary Fig. S5 and Supplementary Table S4). Moreover, both treatments entailed an increase in autophagy, which was observed with the LC3-II and p62 markers. To confirm that the decrease in the proteins of the complex was due to their degradation by the lysosome, we inhibited lysosomal activity with ammonium chloride during treatment with cisplatin. This compound not only inhibits lysosomal enzymes, but also enhances cisplatin cytotoxicity and caspase activity [32]. Under these conditions, the proteins of the complex did not decrease (Fig. 5D and Supplementary Table S3). Not only that, the reduction of FBXW7 levels in knockdown experiments, confirmed by the increase in cyclin E, also prevented the degradation of the proteins of the MRN complex due to cisplatin (Fig. 5E and Supplementary Table S3). Subsequently, we checked whether the observed degradation of MRN proteins led to an increase in the lysosomal localization of the MRN complex elicited by FBXW7. To do this, we analyzed the localization of the MRN complex in lysosomes using light microscopy in U2OS and U2OS::*HA FBXW7ΔF* cells treated with cisplatin and concanamycin A to block autophagic flux. Figure 6 shows that treatment with concanamycin A in U2OS cells (Fig. 6A, left panels, and Fig. 6B) allows to detect a partial localization of NBS1 in the lysosomes, which increases substantially when the cells are treated with cisplatin and concanamycin A (note the arrow). However, the expression of FBXW7ΔF (Fig. 6A, right panels, and Fig. 6B) avoids this localization. Finally, to find out whether overexpression of *FBXW7ΔF*, which reduces MRN degradation (Supplementary Fig. S6), would lead to a decrease in sensitivity to cisplatin, we performed a cell viability assay. We generated a stable doxycycline-inducible U2OS cell line overexpressing *HA FBXW7ΔF* and compared the number of colonies that appeared after cisplatin treatment when it expressed *HA FBXW7ΔF* versus when it did not. Figure 6C shows that FBXW7ΔF increases the viability of cisplatin-treated cells. Namely, the increase of SCF (FBXW7) substrates, among which MRN are included, favors cell viability after DNA damage. Overall, we can conclude that SCF (FBXW7) induces polyubiquitylation and degradation of the MRN complex, especially after suffering catastrophic DNA damage, through the autophagy/lysosome pathway. Under these conditions, FBXW7 enhances cisplatin-induced cell death.

## Discussion

The F-box proteins are the determinants of the specificity of SCF ubiquitin ligase complexes, and, through their substrates, they participate in multiple cellular processes. Specifically, the F-box protein FBXW7 regulates stability of numerous proteins involved in cell-cycle regulation, proliferation, differentiation, and apoptosis. FBXW7 primarily targets oncoproteins, such as cyclin E, PLK1, AURKA, c-MYC, mTOR, NOTCH, MCL1 or c-JUN, but also tumor suppressors such as p53 [24]. Consistent with that, the *FBXW7* gene appears mutated in a wide variety of cancers. In fact, 3.23% of 46 305 human cancers analyzed had mutations in *FBXW7*, especially endometrial cancer, colorectal cancer, cervical cancer, and esophagogastric adenocarcinoma [33].

In this work, we found that endogenous FBXW7 is associated with the MRN complex in normally growing mammalian cells, inducing its degradation through the autophagy/lysosome pathway. Autophagy is a highly selective process that occurs in all eukaryotic cells to recycle defective or useless proteins or organelles. It takes place both under physiological conditions and after being induced by various cellular stresses [34]. Nucleophagy is a subtype of selective autophagy that targets nuclear components. It is involved in maintaining cell homeostasis, at least in some organisms, and when it is disturbed, various pathologies can occur, including cancer [35]. FBXW7 induces the polyubiquitylation of MRN, which allows its association with p62/LC3 and, therefore, its degradation by lysosomes. In fact, FBXW7ΔF, which prevented the ubiquitylation of the MRN complex, largely reduced the interaction of MRN with p62/LC3. To support these results, both MRN and p62/LC3 have a perinuclear location when cells are treated with lysosomal inhibitors, similar to that found when other nuclear components are degraded by lysosomes [36]. Moreover, rapamycin treatment of U2OS cells also reduced the MRN protein complex, as described for the degradation of other autophagic substrates [37]. Taken together, our data reveal that FBXW7 is involved in the regulation of basal levels of the MRN complex. Furthermore, to our knowledge, this would be the first or one of the first physiological types of mammalian nucleophagy described to date [38].

Several reports have implicated FBXW7 in the response to DNA damage, due to ubiquitylation of several key regulatory proteins such as p53, PLK1 or SOX9 [23, 25, 39], and in DNA damage repair. Regarding this last process, FBXW7 promotes K48-linked polyubiquitylation and degradation of BLM, an ATP-dependent RecQ DNA helicase involved in the regulation of HR and NHEJ DSB repair pathways [40]. Moreover, FBXW7 also polyubiquitylates XRCC4, in this case through the K63 linkage, to enhance DNA binding of KU70/KU80 and facilitate NHEJ repair [41]. Now, we provide evidence showing that DNA damage-induced cell death increases the degradation of MRN by the FBXW7/lysosome. We found that in vivo treatment of cells with chemotherapeutic agents such as cisplatin or doxorubicin did not alter the stability of the MRN complex, unless the drug concentrations used induced cell death. These results suggest that, while the damage caused by these agents can be repaired, the MRN complex remains intact to perform its function as a sensor for DNA breaks. In contrast, when damage exceeds the ability of cells to repair DNA, DNA break sensors should be degraded to promote, or at least not interfere, cell death. In fact, the expression of a dominant negative mutant of *FBXW7* reduced cell death after cisplatin treatment, although we cannot guarantee that it is solely due to the greater stability of MRN. In a similar way, some studies have associated cell death with the loss of nuclear KU70 and KU80, or with the cytosolic accumulation of these proteins [42]. It was reported that oxidative stress-induced apoptosis in pancreatic acinar cells caused caspase-dependent degradation of KU proteins and decreased KU binding to nuclear transporters, which subsequently resulted in a reduction in nuclear KU70/KU80 [43]. Conversely, in the early stages of cancer, in which uncontrolled cell proliferation occurs, KU levels and their ability to bind to DNA increase. Repair of high DNA damage would prevent further genomic instability [44]. Other authors correlated the reduction in KU proteins after transient focal cerebral ischemia in mice with apoptotic DNA fragmentation, suggesting that loss of KU and consequent failure of the DNA repair mechanism could contribute to DNA fragmentation [45]. Gama et al, however, showed that apoptosis induced by different drugs increased ubiquitylation and degradation of KU70 through the proteasome [46]. On the basis of these findings, it is conceivable that MRN degradation via the autophagy/lysosomal pathway may contribute to DNA damage-induced cell death.

Overexpression of the MRN complex has been reported to be associated with the appearance of cisplatin-resistant tumor cells, leading to a poor prognosis [47]. Therefore, the importance of finding compounds that prevent the repair of damage caused by cisplatin is highlighted to avoid the appearance of resistant cells and induce the death of tumor cells. In fact, for years there have been attempts to find an inhibitor or combination of inhibitors that allows the elimination of these tumor cells with some encouraging results [48, 49]. Based on our results showing that induction of apoptosis after DNA damage increases MRN degradation, we could suggest that if we were able to increase the specific degradation of MRN by FBXW7/lysosome after cisplatin treatment, tumor cells could be eliminated, preventing the appearance of resistance to genotoxic drugs. A small molecule could be designed to enhance MRN degradation, similar to those described previously [50]. Furthermore, since it is a physiological degradation, perhaps a lower dose of cisplatin could degrade MRN in the presence of that compound, thus preventing drug toxicity in the patient. However, further studies are required to probe this idea.

## Materials and methods

### Plasmids, cloning, and sequencing

pCDNA3 Flag FBXW7, pCMVHA FBXW7, and pCMVHA FBXW7ΔF have been previously described [25, 51]. pCMV6 Flag MRE11 and pRG4 Myc Ub were from Origene (Rockville, MD, USA) and ATCC (Manassas, VA, USA), respectively. pCMVHA RAD50, pCMVHA NBS1, pLenti 6.3 HA FBXW7, pLenti 6.3 HA FBXW7ΔF, pLenti 6.3 HA βTrCP and pCDNA5 FRT/TO-HA FBXW7ΔF were obtained by cloning the corresponding PCR fragments in pCMVHA [52] or pLenti 6.3 or pCDNA5 FRT/TO from Addgene (Watertown, MA, USA). The sequences of the constructs were verified on both strands with an automatic sequencer.

### Viral transductions

Recombinant lentivirus expressing HA FBXW7, HA FBXW7ΔF or HA βTrCP were produced in the HEK293T cell line. Cells were transfected using Xfect reagent (Clontech, Mountain View, CA, USA) with the following plasmids: one derivative of pLenti6.3 as described above and pMD2-G, pMDLg-pRRE and pRSV-Rev (Addgene). After 48 h of transfection, the cell supernatant was harvested and passed through a 0.45 μm Puradisc 25 mm filter (GE Healthcare, Little Chalfont, UK). Subsequently, the viruses were concentrated using a Lenti-X concentrator (Clontech). Cells were transduced with supernatant containing lentivirus for 24 h, and transduced cells were selected after seven days in 20 μg/ml blasticidin (Gibco, Thermo Fisher Scientific, Waltham, MA, USA).

### Cell culture, transient and stable transfections, drugs, and cell lysis

Routinely, COS-7, U2OS, A549, HeLa, and HEK293T cells (from ATCC) and derivatives were grown in Dulbecco’s modified Eagle’s medium (DMEM) from BioWest, Nuaillé, France as described [53]. The COS-7, U2OS, HeLa and HEK293T cell lines were recently authenticated by STR profiling. DLD1 and DLD1 *FBXW7*^−/−^ cells [54] were grown in McCoy’s 5A medium (BioWest) supplemented with 10% (v/v) foetal calf serum (FCS), 100 μg/ml streptomycin and 100 U/ml penicillin from Gibco. A U2OS cell line with inducible expression of *HA FBXW7ΔF* was also constructed as previously described [55] using pCDNA5 FRT/TO-HA FBXW7ΔF, pOG44 (Addgene) and the U2OS Flp In T-REx host cell line [56]. 72 h after transfection, cells were selected in DMEM with 10% tetracycline-free foetal bovine serum (Gibco) supplemented with 100 μg/ml of hygromycin (Gibco). The expression of the resulting foci was tested. Gene expression was induced by the addition of 2 μg/ml doxycycline (DOX, Sigma-Aldrich, St. Louis, MO, USA) for 24 h. Serum starvation was obtained by growing HeLa cells for 3 days in medium supplemented with 0.15% FCS. The DNA constructs were transiently transfected by electroporation or by using a lipid transfection reagent (Xfect, Clontech). Cells were harvested and lysed 18 h or 48 h after transfection, respectively. Where appropriate, transfected cells were retransfected with other plasmids. In the indicated experiments, cells were treated with cisplatin (CDDP, Sigma-Aldrich), doxorubicin (Dx, Sigma-Aldrich), MG132 (20 μM, Santa Cruz Biotechnology, Dallas, TX, USA), ammonium chloride (40 mM, Sigma-Aldrich), concanamycin A (Con A, 50 nM, Sigma-Aldrich), rapamycin (10 μM, Sigma-Aldrich) and trehalose (100 mM, Sigma-Aldrich), and harvested at various times.

Whole cell extracts were prepared at 4 °C in 420 mM NaCl, 50 mM Tris-HCl (pH 7.5), 0.1% sodium dodecyl sulfate (SDS), 0.5% sodium deoxycholate, 1% Nonidet P-40 (NP40), 10% glycerol, 1 mM phenylmethylsulfonyl fluoride (PMSF), 1 μg/ml aprotinin, 1 μg/ml pepstatin, 1 μg/ml leupeptin and 10 μg/ml chymostatin for 20 min, and sonicated. The extracts were centrifuged at 20,000 *g* for 20 min and the supernatants were frozen in liquid nitrogen and stored at −80 °C. Protein concentration was determined using the Bradford assay (Bio-Rad, Hercules, CA, USA).

### Electrophoresis, Western blot analysis, and antibodies

Proteins were separated by SDS-polyacrylamide gel electrophoresis (SDS-PAGE) and gels were electroblotted onto nitrocellulose membranes and probed with the following antibodies: anti-HA-peroxidase monoclonal antibody (#12013819001, Roche, Basel, Switzerland); anti-cyclin E (sc-377100), anti-MRE11 (sc-135992), anti-BrCA1 (sc-6954), anti-histone H1 (sc-393358), anti-α actinin (sc-17829) and anti-p53 (sc-126) monoclonal antibodies, and anti-MCL1 (sc-819) polyclonal antibody (Santa Cruz Biotechnology); anti-α tubulin (T9026), anti-β actin (A5441) and anti-Flag (F3165) monoclonal antibodies, and anti-p62 (SAB5701338) polyclonal antibody (Sigma-Aldrich); anti-PARP (#551024) and anti-cyclin B (#610220) monoclonal antibodies (BD Biosciences, Franklin Lakes, NJ, USA); anti-PLK1 (#05-844) and anti-γH2AX (#05-636) monoclonal antibodies (EMD Millipore, Merck, Darmstadt, Germany); anti-cleaved caspase-3 (#9664), anti-NBS1 (#14956) and anti-K48 (#8081) polyubiquitin monoclonal antibodies (Cell Signaling Technology, Danvers, MA, USA); anti-RAD50 monoclonal antibody (GTX70228, GeneTex, Irvine, CA, USA); and anti-LC3 (NB100-2220) and anti-LAMP1 (NB120-19294) polyclonal antibodies (Novus Biologicals, Littleton, CO, USA). Peroxidase-coupled donkey anti-rabbit IgG (NA934V) and sheep anti-mouse IgG (NA931) were obtained from GE Healthcare. Immunoreactive bands were visualized using the Enhanced Chemiluminescence Western blotting system (ECL, GE Healthcare).

Protein level quantification was carried out using ImageJ software (Image Processing and Analysis in Java; National Institutes of Health, Bethesda, MD, USA; http://imagej.nih.gov/). Original Western blots for all relevant figures are shown in “Supplementary Data/Original blots”.

### Co-immunoprecipitation experiments

1–2 mg of protein from cytosolic and nuclear fractions or NP40 extracts (420 mM NaCl, 10 mM Tris-HCl (pH 7.5), 1% NP40, 10% glycerol, 1 mM PMSF, 1 μg/ml aprotinin, 1 μg/ml pepstatin and 1 μg/ml leupeptin, centrifuged and then diluted to 150 mM NaCl) were incubated with normal mouse (sc-2025) or rabbit (sc-2027) sera (Santa Cruz Biotechnology) for 30 min and subsequently with protein G or A-sepharose beads (GE Healthcare), respectively, for 1 h at 4 °C. After centrifugation, the beads were discarded and the supernatants incubated for 2 h with anti-FBXW7 (A301-720A, Bethyl laboratories, Montgomery, TX, USA), anti-Flag (F3165, Sigma-Aldrich), anti-MRE11 (#4847, Cell Signaling Technology), anti-NBS1 (#14956, Cell Signaling Technology), anti-RAD50 (NB110-60483, Novus Biologicals) or with normal mouse or rabbit sera, followed by protein G or A-sepharose beads for 1 h. The beads were washed, and the bound proteins were solubilized by adding SDS sample buffer and heated at 95 °C for 5 min.

### Subcellular fractionation

Cytosolic extracts (S100) and nuclear extracts (NE) were prepared as described [57].

The lysosome-enriched cellular fraction was obtained as previously described [58]. Briefly, cells were resuspended in hypotonic buffer (40 mM KCl, 5 mM MgCl_2_, 2 mM EGTA, 10 mM HEPES, pH 7.5) for 30 min on ice. Later, they were homogenized by shearing through a 28.5-gauge needle 30 times. After centrifugation at 1000 *g* for 10 min, the supernatant was centrifuged again at 12,000 *g* for 10 min. This second supernatant was the cytosolic fraction, while the pellet, enriched for the lysosome, was further washed in an isotonic buffer (150 mM NaCl, 5 mM MgCl_2_, 2 mM EGTA, 10 mM HEPES pH 7.5) and dissolved in a lysis buffer (1% Triton X-100, 150 mM NaCl, 50 mM Tris-HCl pH 7.5) for further analysis.

### Knockdown experiments

Cells were transfected with FBXW7α-, LC3- or p62-siRNA [58, 59] using the Oligofectamine method (Invitrogen, Carlsbad, CA, USA) to suppress the expression of endogenous genes. EGFP-siRNA [58] was used as a non-specific control. Cells were harvested 48 h after transfection and the reduction in protein levels was confirmed by Western blotting.

### In vitro and in vivo ubiquitylation assays

In vitro ubiquitylation was performed in a volume of 10 μl containing 50 mM Tris-HCl (pH 7.6), 5 mM MgCl_2_, 0.6 mM DTT, 2 mM ATP, 0.5 μg recombinant SCF (FBXW7) complex expressed in Sf21 insect cells, 1.5 ng/μl E1 (His_6_-ubiquitin-activating enzyme, Boston Biochem, Cambridge, MA, USA), 10 ng/μl His_6_-UbcH3 (E2, Boston Biochem), 10 ng/μl UbcH5a (E2, Boston Biochem), 2.5 μg/μl ubiquitin (Sigma-Aldrich), 1 μM ubiquitin aldehyde (Boston Biochem), and 1 μl [^35^S]-methionine-labelled in vitro transcribed/translated MRE11, NBS1 or RAD50, as substrate. The reactions were incubated at 30 °C for 1 h and analyzed by SDS-PAGE and autoradiography.

In vivo ubiquitylation experiments were performed in HEK293T cells transfected with the indicated plasmids and treated with ammonium chloride 4 h before harvesting. Cells were washed in phosphate-buffered saline (PBS), lysed at 95 °C for 15 min in NP40 buffer supplemented with 5% SDS and 10 mM iodoacetamide, and then diluted four times in NP40 buffer supplemented with 10 mM iodoacetamide. Epitope-tagged MRE11, NBS1, or RAD50 were immunoprecipitated and proteins separated by SDS-PAGE, electroblotted, and probed with different antibodies.

### AnnexinV binding assays

U2OS cells were treated with CDDP or Dx for 24 h. One million cells were washed in cold PBS and resuspended in 100 μl of annexin V binding buffer containing 5 μl propidium iodide and 5 μl of annexin V-FITC (Annexin V-FITC apoptosis detection kit I, BD Biosciences), incubated for 15 min at room temperature in the dark and diluted in 400 μl of annexin V binding buffer. Fluorescence was measured on a FACSCanto II cytometer (BD Biosciences) within 1 h. Cell populations (viable, early apoptotic, and late apoptotic) were identified by measuring fluorescence on FITC-A and PerCP-Cy5-5-A channels. Annexin V binding assays were repeated three times in independent experiments. Data were analyzed using Diva software.

### Immunofluorescence microscopy

Cells were grown on coverslips, fixed in −20 °C 100% methanol for 10 min on ice, and permeabilized with 0.25% Triton X-100 for 10 min at room temperature (RT). Cells were incubated with blocking buffer (1% BSA, 0.1% Tween 20 in PBS) for 1 h at RT. Coverslips were incubated with primary antibodies for 1 h at RT, washed three times with 1% PBS and once with blocking buffer, and incubated with the appropriate fluorescent secondary antibody for 1 h at RT. The coverslips were then washed three times with 1% PBS and mounted on microscopy slides using mounting reagent with DAPI (Ibidi GmbH, Gräfelfing, Germany). Staining was analyzed using a Leica DMi8 inverted microscope with a 63x oil-immersion objective using the same laser parameters. All microscope images were analyzed with ImageJ software. Primary antibodies used: anti-RAD50 mouse monoclonal antibody (GTX70228, GeneTex), anti-NBS1 rabbit monoclonal antibody (#14956, Cell Signaling Technology), anti-MRE11 mouse monoclonal antibody (sc-135992, Santa Cruz Biotechnology), anti-p62 rabbit polyclonal antibody (NBP1-49955, Novus Biologicals), anti-LC3 rabbit polyclonal antibody (NP100-2220, Novus Biologicals) and anti-LAMP1 mouse monoclonal antibody (sc-20011, Santa Cruz Biotechnology).

### Cell viability assays

Briefly, U2OS Flp In T-REx HA FBXW7ΔF cells were induced or not by adding 2 μg/ml DOX for 24 h, and then treated with CDDP (30 μM) for 4 h and washed. After 24 h, 5 × 10^3^ and 10^4^ cells of each condition were seeded in 6-well plates and incubated for 8–12 days. Subsequently, colonies were stained with 0.5% crystal violet solution in methanol for 20 min, washed, and air-dried. Stained colonies were counted using ImageJ software. All assays were carried out in triplicate.

### Quantification and statistical analysis

Statistical details of the experiments (number of replicates and use of standard deviation) are included in the Figure Legends. Statistical analyses were performed with unpaired Student’s *t*-tests. Significance is indicated in the figures as *p < 0.05, **p < 0.01, ***p < 0.001 and ****p < 0.0001.

## Supplementary information

Supplementary figure legends

Supplementary Figure S1

Supplementary Figure S2

Supplementary Figure S3

Supplementary Figure S4

Supplementary Figure S5

Supplementary Figure S6

Supplementary Table S1

Supplementary Table S2

Supplementary Table S3

Supplementary Table S4

Origunal blots

Reproducibility checklist

## Data Availability

Data sharing not applicable to this article as no datasets were generated or analyzed during the current study.
