# Supplementary material for: Cisplatin-induced cell death increases the degradation of the MRE11-RAD50-NBS1 complex through the autophagy/lysosomal pathway
Source: Cell Death Differ. 2022 Dec 8;30(2):488–99. doi: 10.1038/s41418-022-01100-1 (PMC9950126; doi:10.1038/s41418-022-01100-1)
Supplement: Supplementary file 1 — Supplementary figure legends [file 41418_2022_1100_MOESM1_ESM.docx]

**SUPPLEMENTARY FIGURE LEGENDS**

**Supplementary Figure S1.** Effect of lysosomal and proteasomal inhibitors on the degradation of RAD50, NBS1 and MRE11 mediated by SCF (FBXW7).

**A.** Identification of MRE11 as a new FBXW7 interacting protein. Tandem mass spectrometry spectrum of a single peptide corresponding to the MRE11 protein. The diagram shows fragment ions corresponding to main fragmentation series (b-amino and y-carboxy). **B.** NP40 or nuclear extracts from monkey COS-7 cells and human A549 cells, respectively, were used to immunoprecipitate endogenous FBXW7, and the obtained complexes were analyzed by immunoblotting. IgG: immunoprecipitation using normal rabbit serum, as a control. Inp: the input lane was loaded with 1/20 of the extract. Western blot against PLK1 was used as a control of the efficiency of FBXW7 immunoprecipitation. The data are representative of three independent experiments. **C.** U2OS cells were treated with MG132 for the indicated times, whole cell extracts prepared, and membranes immunoblotted with the indicated antibodies. C: extracts of untreated cells. Data are representative of at least two independent experiments. **D.** The graphs show the quantification of BrCA1 and MRN levels from figure 1D using ImageJ software. **E.** The graph shows the quantification of MRN and cyclin E levels from figure 1E using ImageJ software. **F.** The graph shows the quantification of MRN levels from figure 1F using ImageJ software. Error bars represent the SD (n = 3). *p < 0.05, **p < 0.01 ***p < 0.001, ****p < 0.0001 (Student’s *t* test).

**Supplementary Figure S2.** Expression level of MRN complex proteins in DLD1 *FBXW7^-/-^* cells.

**A.** Whole cell extracts from DLD1 and DLD1 *FBXW7^-/-^* cells were prepared, transferred and blotted with the indicated antibodies. **B.** The graph shows the quantification of MRN levels from the supplementary figure S2A using ImageJ software. Error bars represent the SD (n = 3). ***p < 0.001 (Student’s *t* test).

**Supplementary Figure S3.** MRE11, NBS1, and RAD50 have the same localization in normally growing U2OS cells, and are localized in the nuclear periphery, along with LC3 and p62, after Con A treatment.

**A.** U2OS cells were grown on coverslips, fixed and incubated with the indicated antibodies, as described in Materials and Methods. Staining was analyzed using a Leica DMi8 inverted microscope with a 63x oil-immersion objective using the same laser parameters. All microscope images were analyzed with ImageJ software. The bars represent 10 μm. **B.** Quantification of U2OS cells treated or not with Con A for 8 h showing RAD50 and LC3 in the nuclear periphery. NP: nuclear periphery. Error bars represent the SD (n = 3). ****p < 0.0001 (Student’s *t* test). **C.** Similar to B, but quantifying the percentage of cells showing MRE11 and p62 labeling at the nuclear periphery. **D.** Comparison of the levels of the proteins of interest in the nuclear extracts of cells U2OS, U2OS::*HA FBXW7* and U2OS::*HA FBXW7ΔF* treated with ammonium chloride used in the experiment of Figure 3C.

**Supplementary Figure S4.** Comparison of the percentage of U2OS versus U2OS::*HA FBXW7ΔF* cells treated with Con A displaying NBS1 in lysosomes.

**A, B, C.** Ponceau staining from filters used in figures 4A, B, C. Images show the similar load level of the lanes of each fraction. **D.** Quantification of Con A-treated U2OS and U2OS::*HA FBXW7ΔF* cells showing NBS1 in lysosome visualized with anti-LAMP1. Error bars represent the SD (n = 3). ****p < 0.0001 (Student’s *t* test).

**Supplementary Figure S5.** Doxorubicin-induced cell death increases the degradation of the MRN complex.

**A.** U2OS cells were incubated with different concentrations of doxorubicin (Dx) for 24 h. Whole cell extracts were analyzed by immunoblotting. Western blots are representative of two replicates. **B.** Percentage of annexin V positive U2OS cells treated as described before were detected by flow cytometry. Error bars represent the SD (n = 3). *p < 0.05, **p < 0.01 (Student’s *t* test).

**Supplementary Figure S6.** Expression level of MRN complex proteins in U2OS Flp In T-REx *HA FBXW7ΔF*.

Whole cell extracts from U2OS Flp In T-REx *HA FBXW7ΔF* treated or not with doxycycline (DOX, 2 μg/ml) for 24 h were analyzed by Western-blotting.
