# Supplementary figures and images for: Cisplatin-induced cell death increases the degradation of the MRE11-RAD50-NBS1 complex through the autophagy/lysosomal pathway
Source: Cell Death Differ. 2022 Dec 8;30(2):488–99. doi: 10.1038/s41418-022-01100-1 (PMC9950126; doi:10.1038/s41418-022-01100-1)

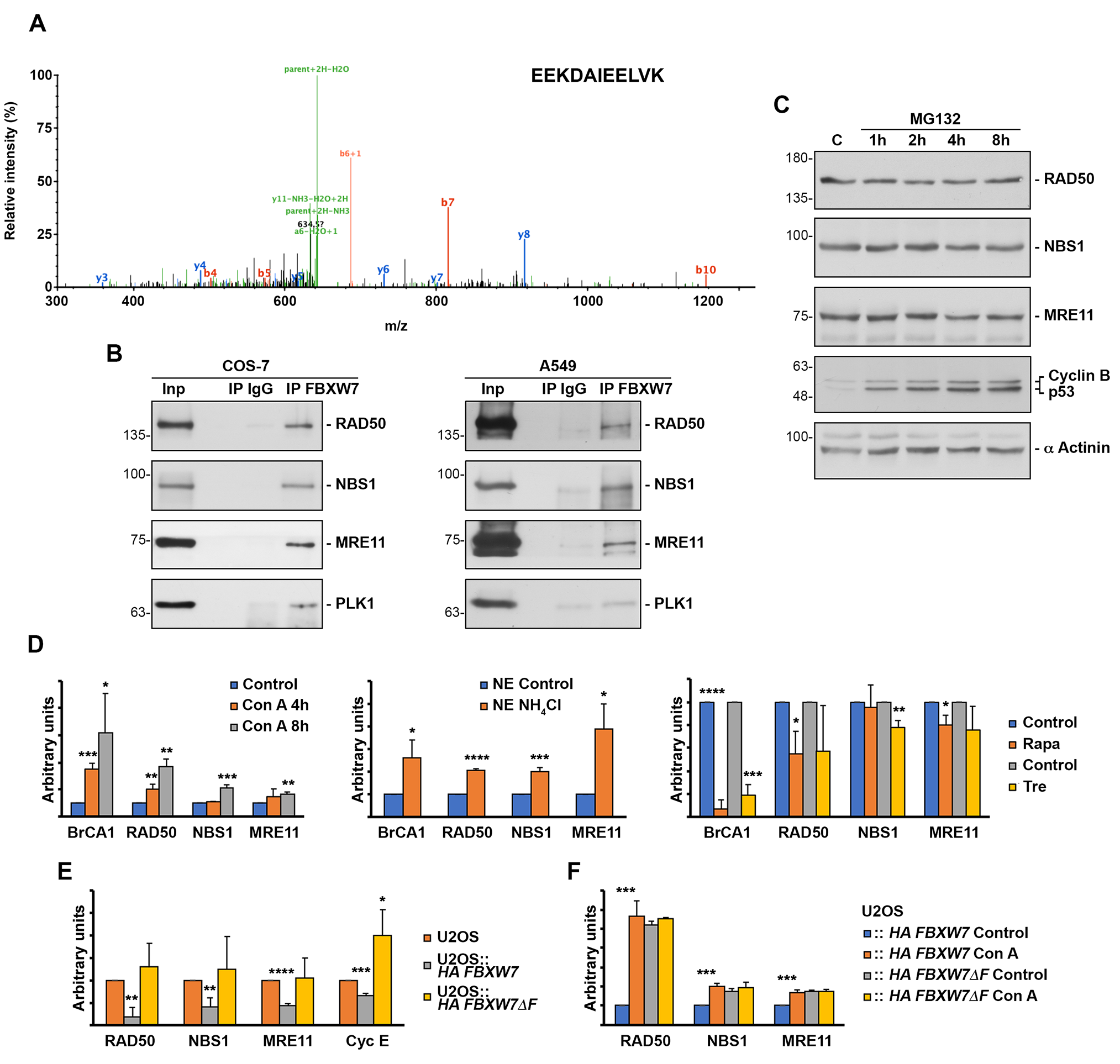

Supplement: Supplementary file 2 — Supplementary Figure S1 [file 41418_2022_1100_MOESM2_ESM.tif]

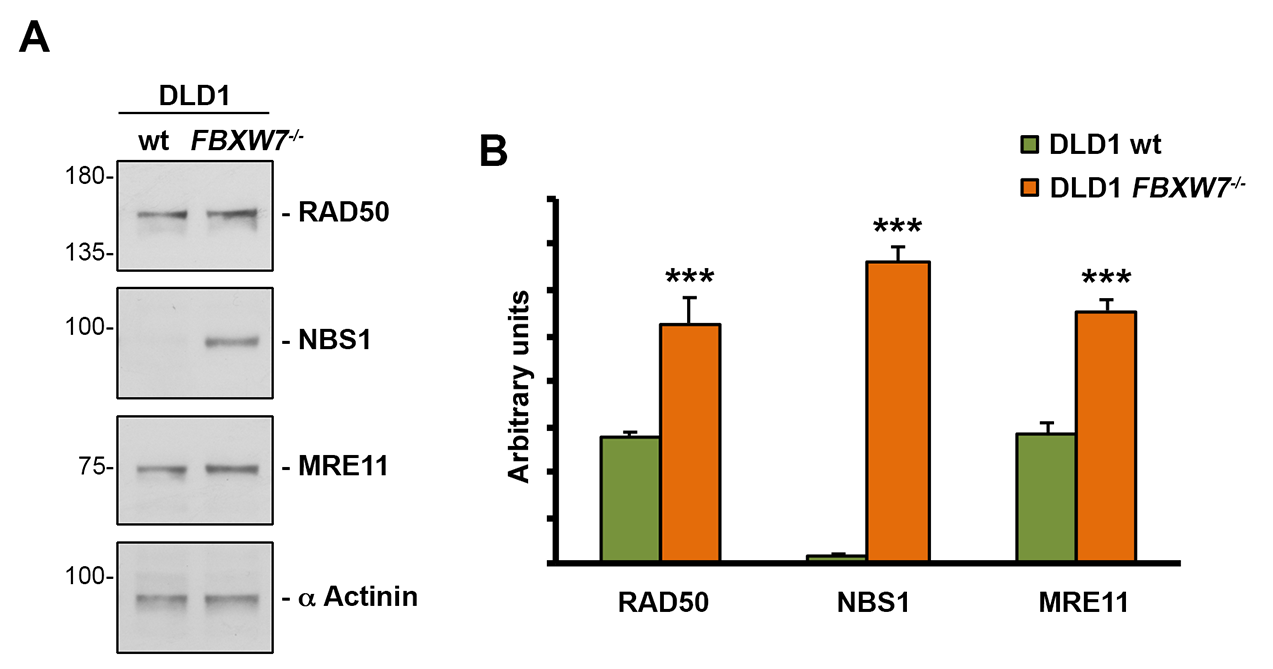

Supplement: Supplementary file 3 — Supplementary Figure S2 [file 41418_2022_1100_MOESM3_ESM.tif]

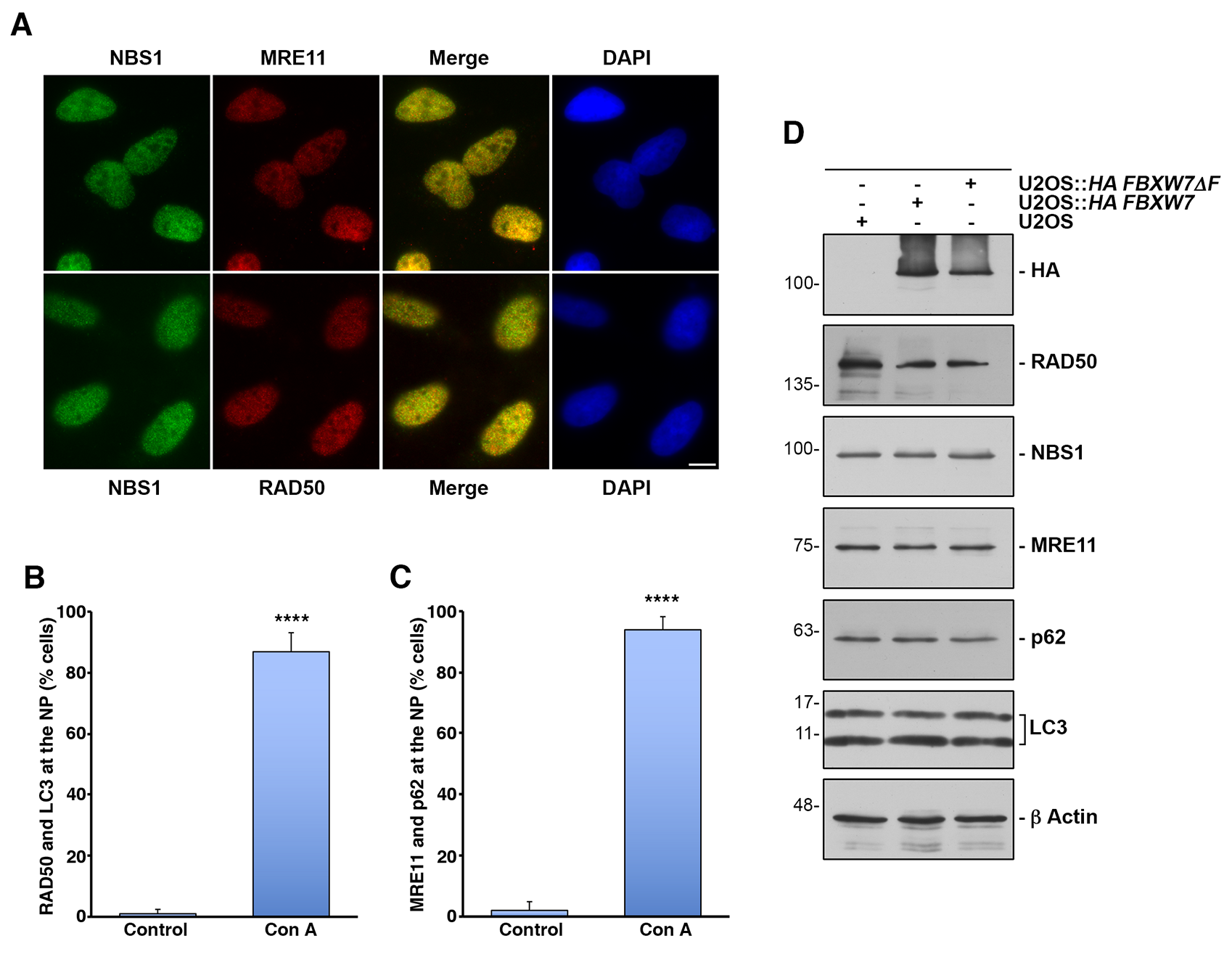

Supplement: Supplementary file 4 — Supplementary Figure S3 [file 41418_2022_1100_MOESM4_ESM.tif]

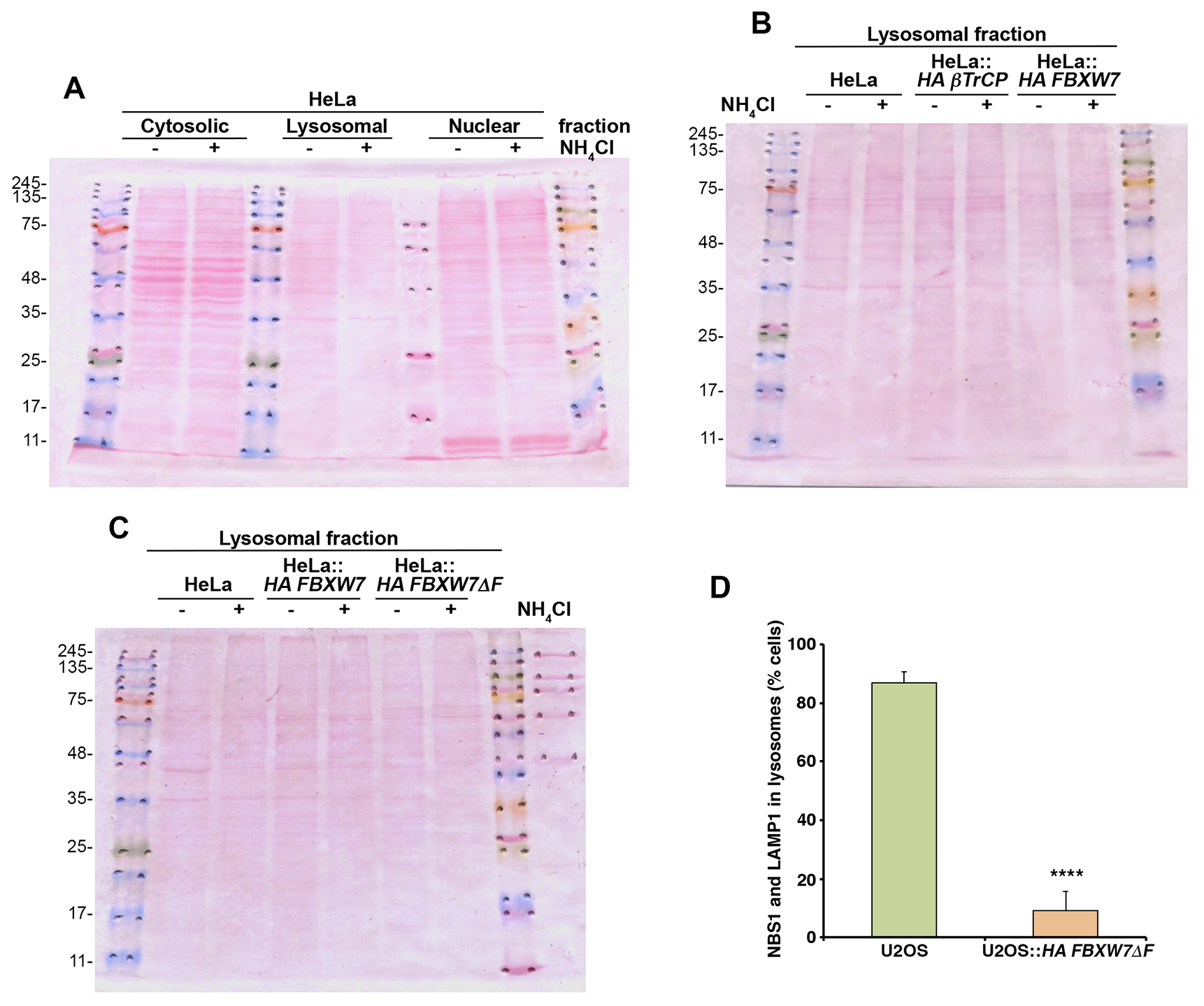

Supplement: Supplementary file 5 — Supplementary Figure S4 [file 41418_2022_1100_MOESM5_ESM.tif]

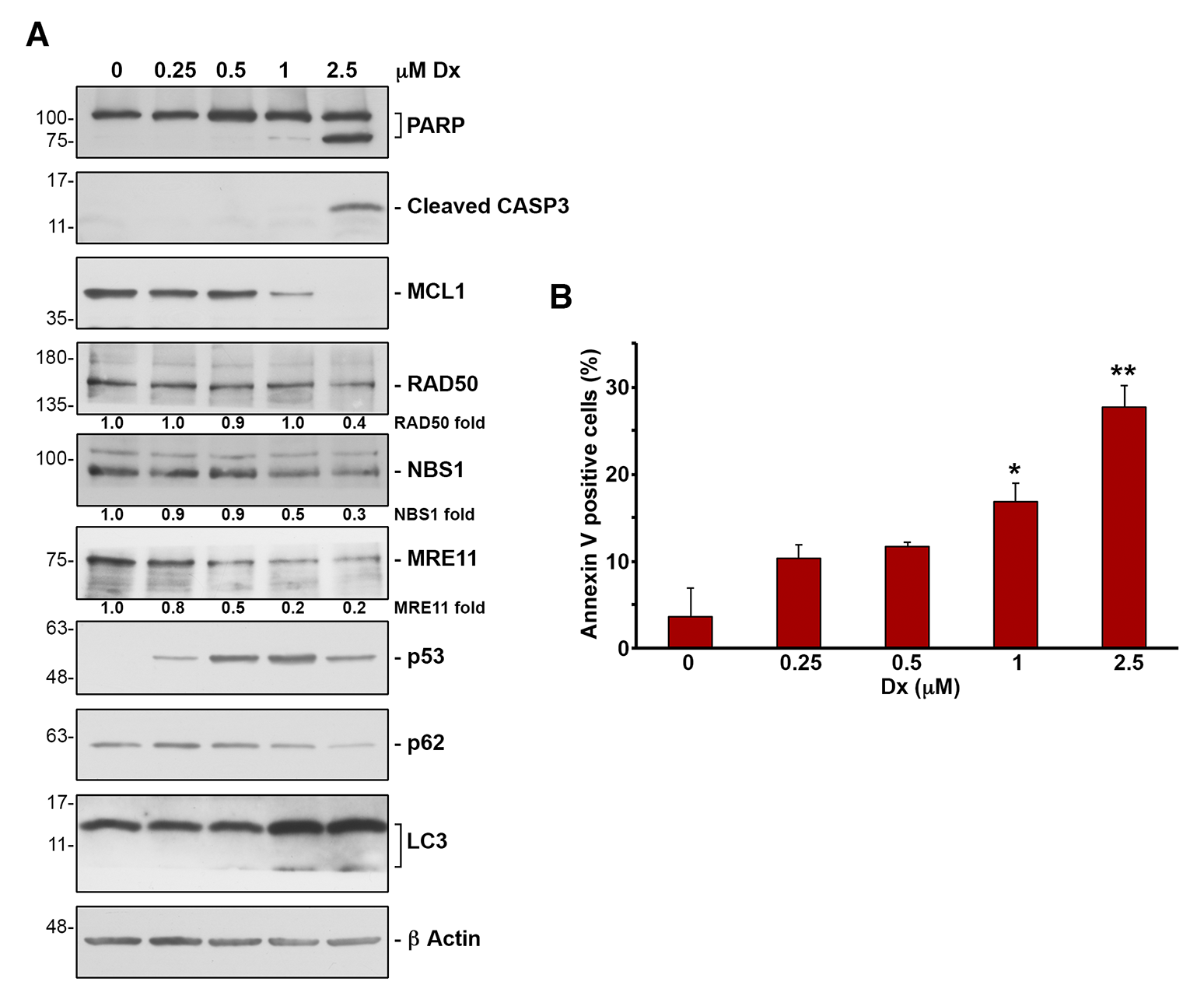

Supplement: Supplementary file 6 — Supplementary Figure S5 [file 41418_2022_1100_MOESM6_ESM.tif]

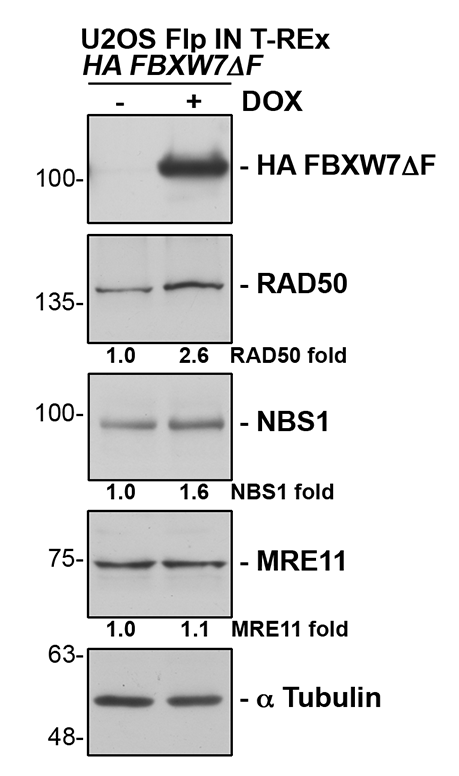

Supplement: Supplementary file 7 — Supplementary Figure S6 [file 41418_2022_1100_MOESM7_ESM.tif]
