# Supplementary material for: Cisplatin-induced cell death increases the degradation of the MRE11-RAD50-NBS1 complex through the autophagy/lysosomal pathway
Source: Cell Death Differ. 2022 Dec 8;30(2):488–99. doi: 10.1038/s41418-022-01100-1 (PMC9950126; doi:10.1038/s41418-022-01100-1)

Figure 1A

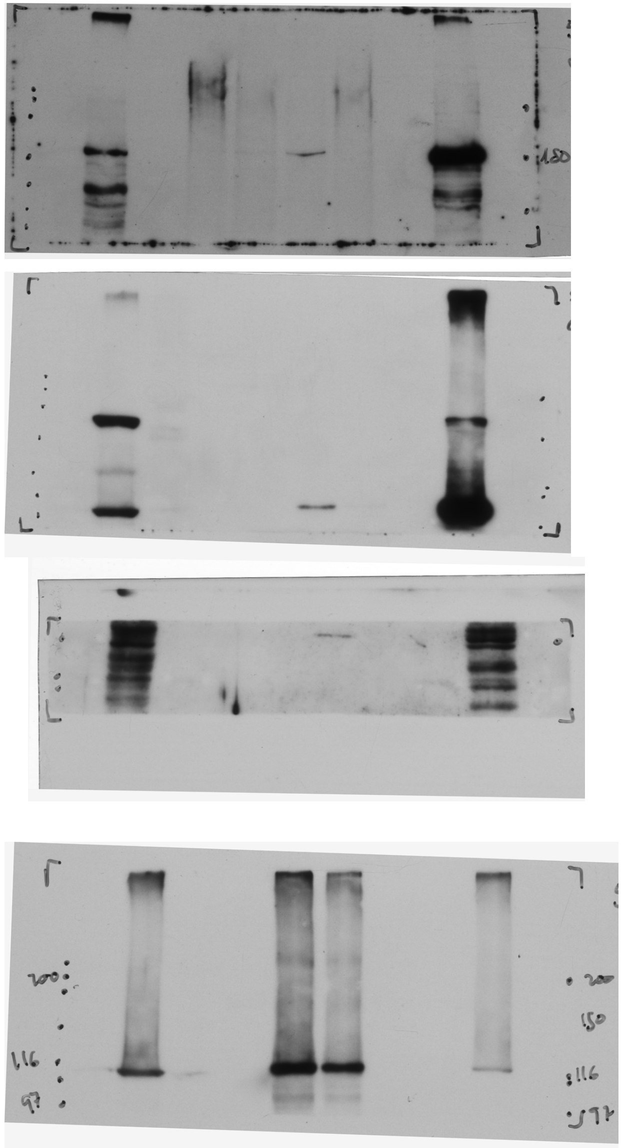

Figure 1B

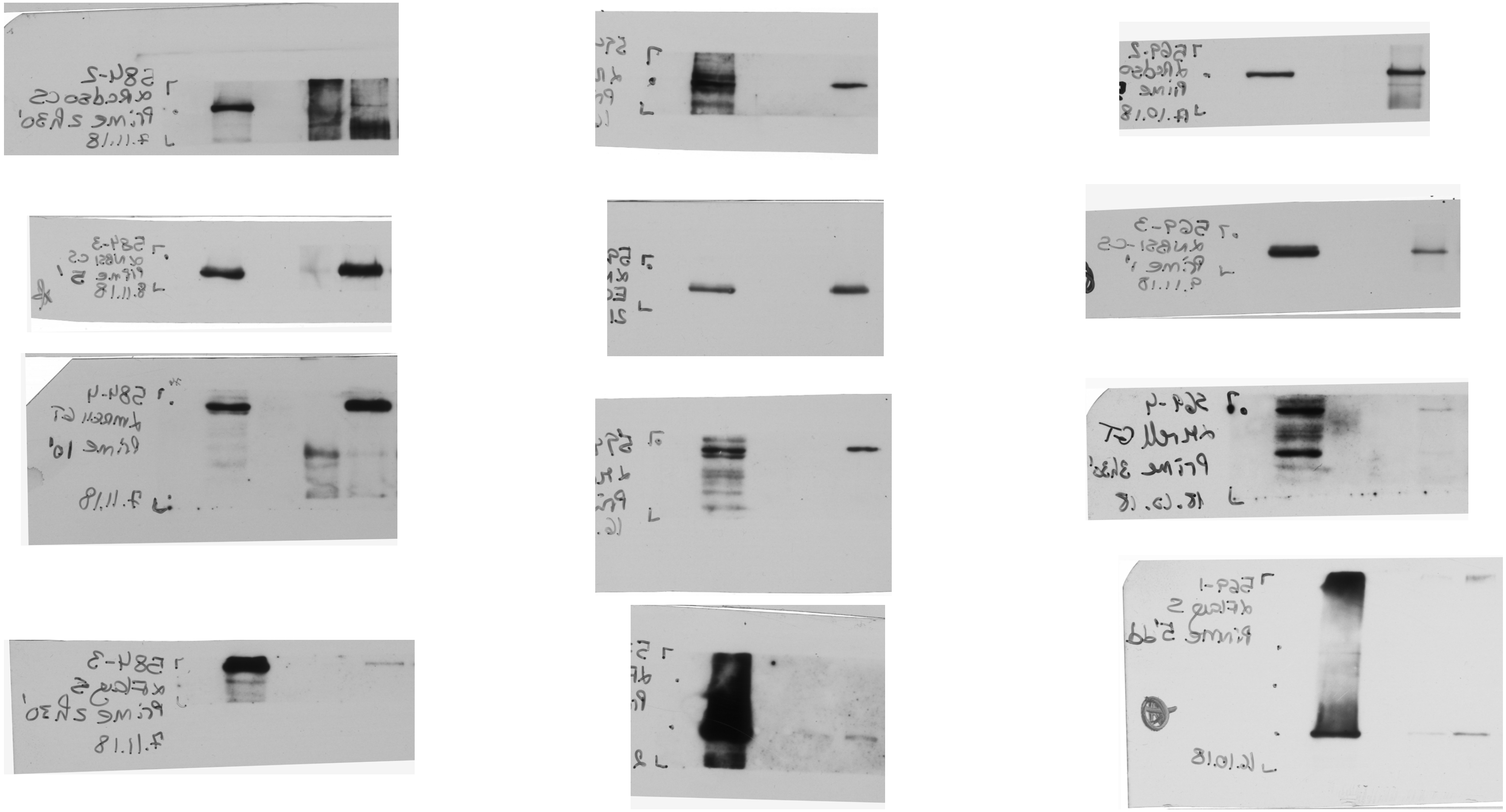

Figure 1C

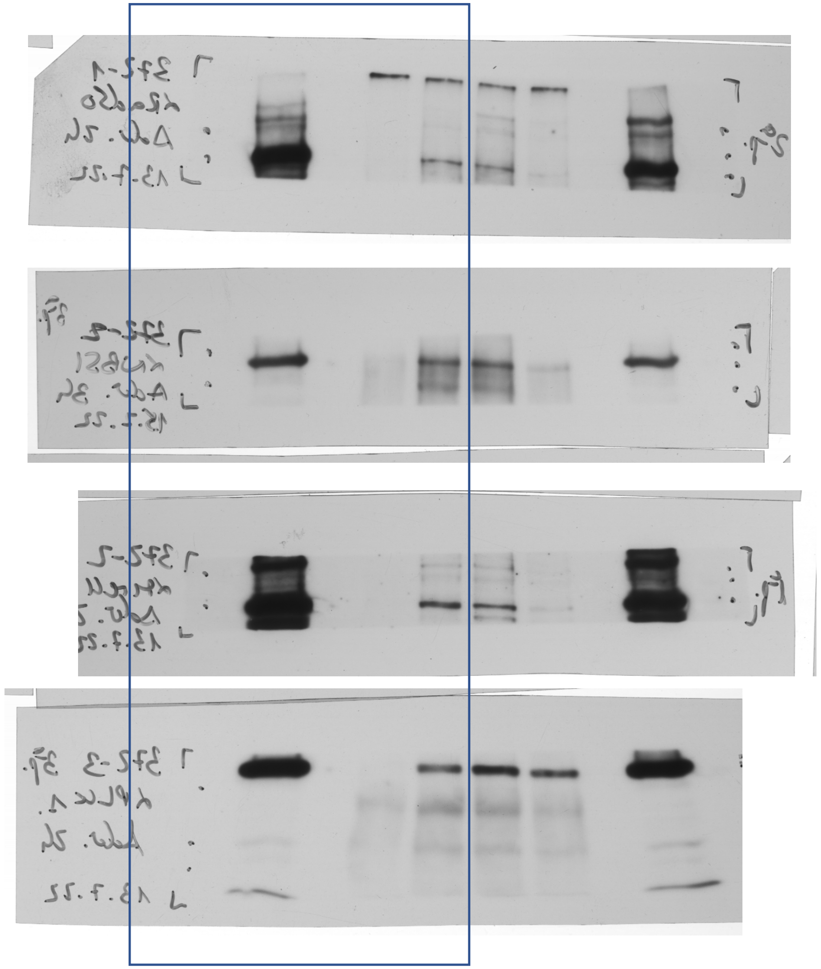

Figure 1D

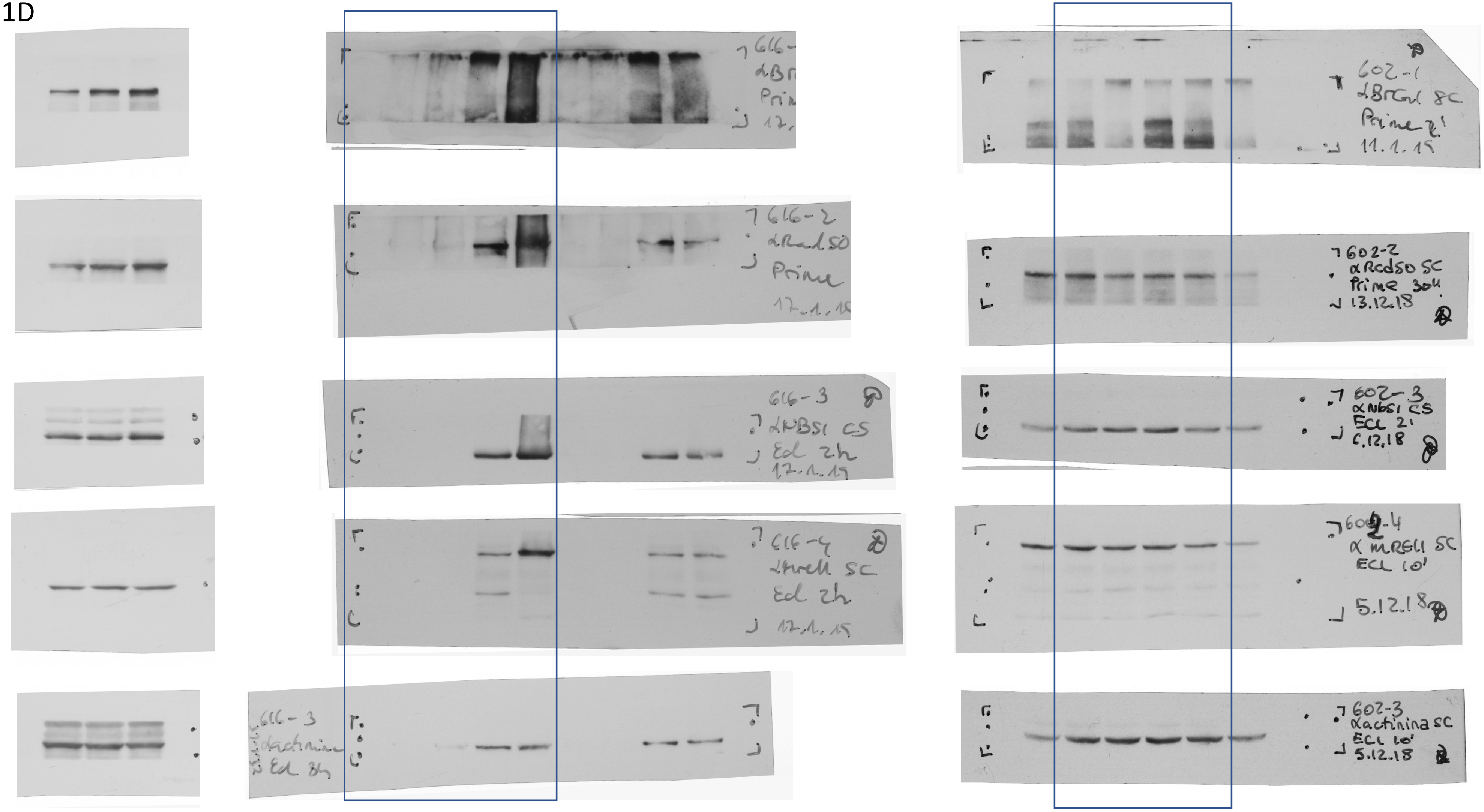
616-2
616-3
616-4
602-1
11.1.19
602-2
αRad50 SC
13.12.18
602-3
αNbs1 CS
602-4
αMRE11 SC
5.12.18
αactinina SC
5.12.18

Figure 1E

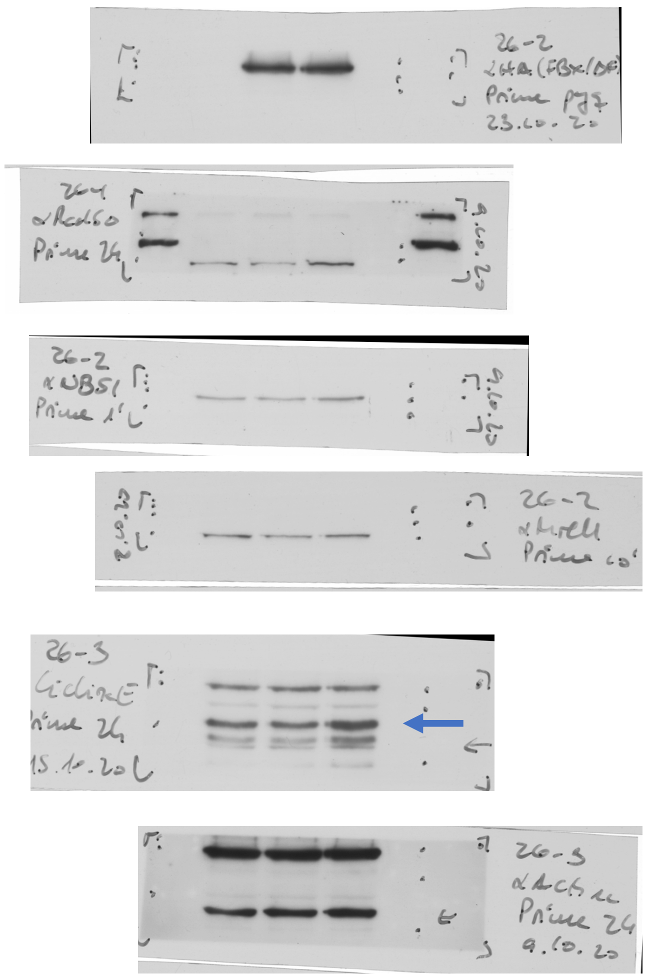

Figure 1F

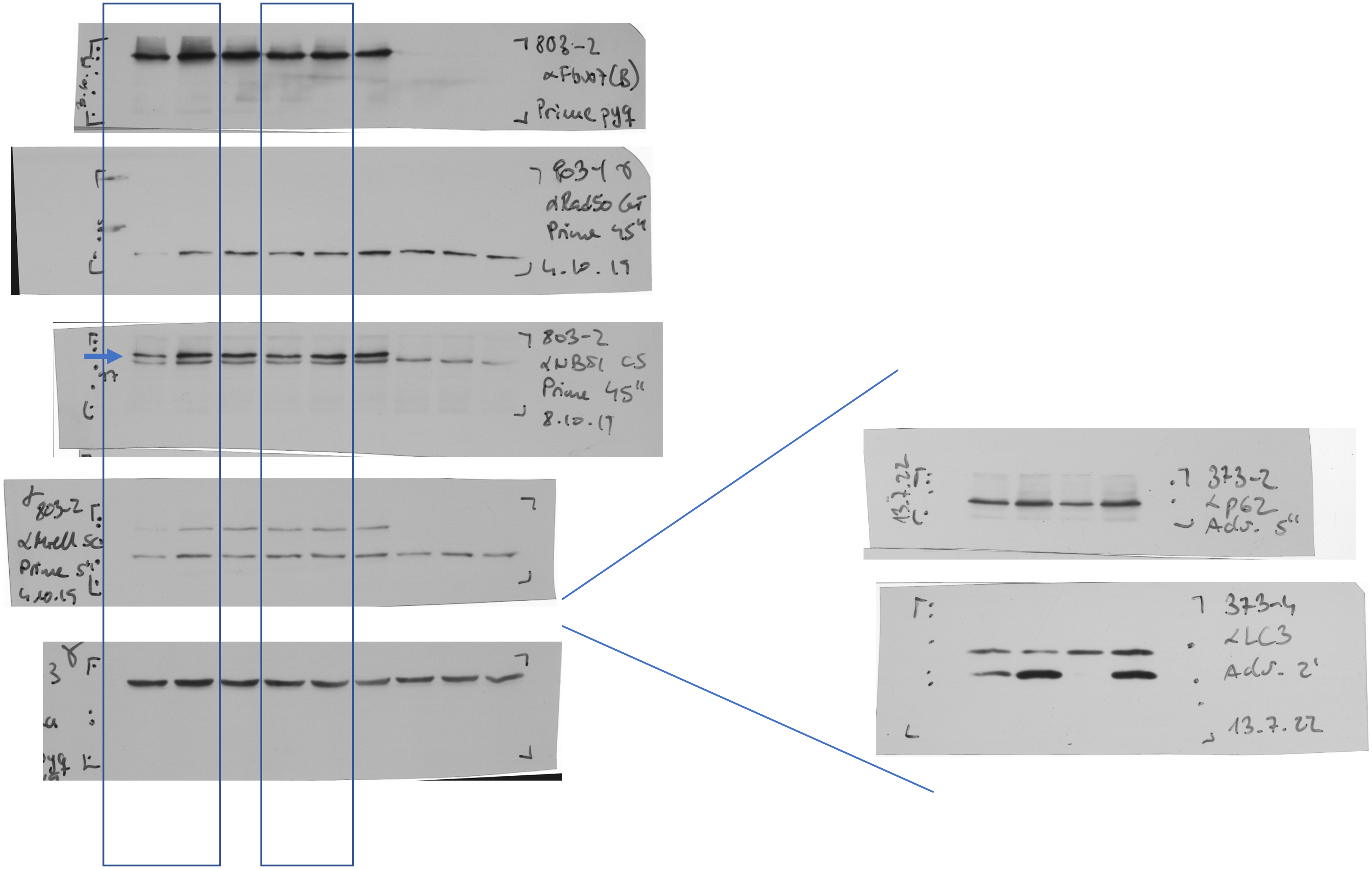
803-2
αFbxo7(B)
Prime p49
803-1
αRad50 GT
Prime 45''
4.10.19
803-2
αNBS1 CS
Prime 45''
8.10.19
803-2
αMre11 SC
Prime 5''
4.10.19
373-2
αp62
Adv. 5''
13.7.22
373-4
αLC3
Adv. 2'
13.7.22

Figure 2A

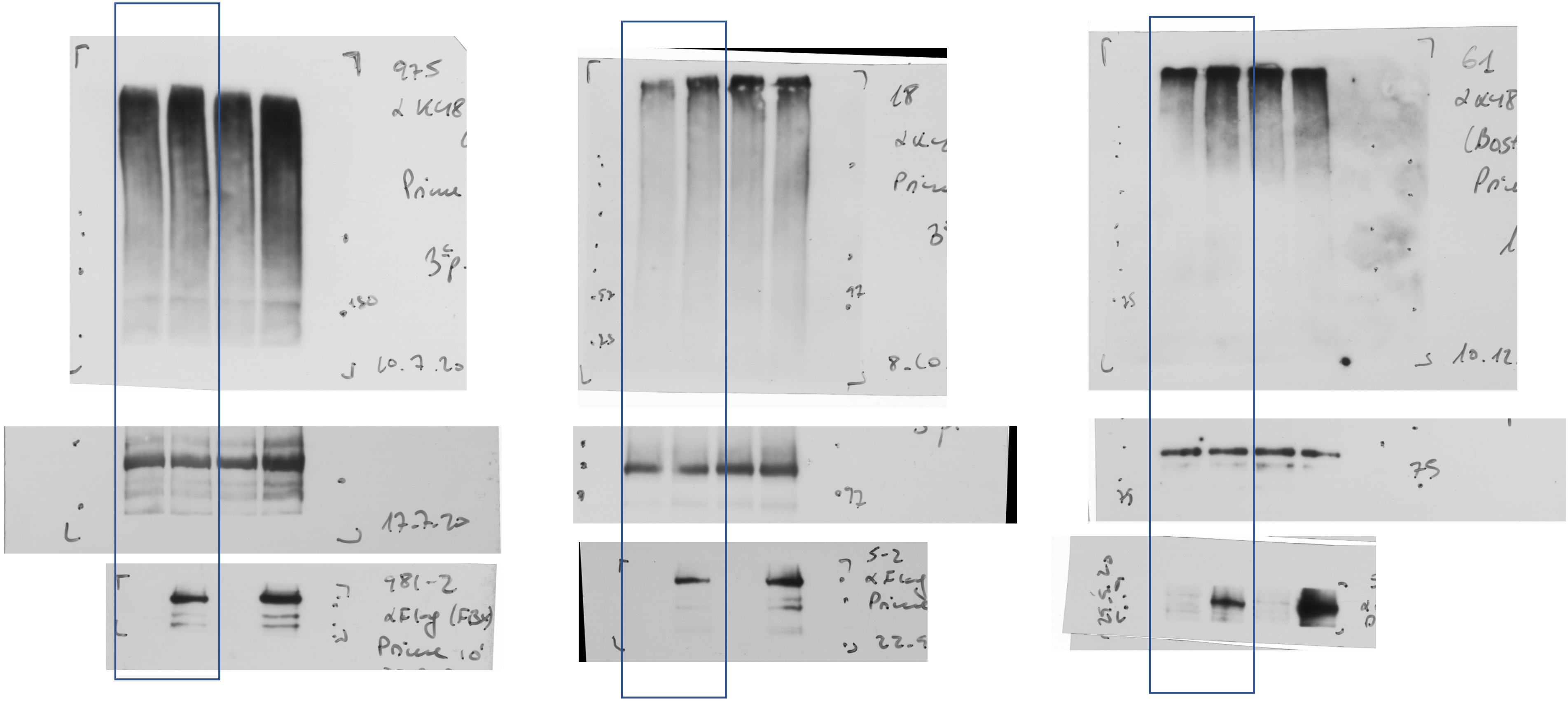

Figure 2B

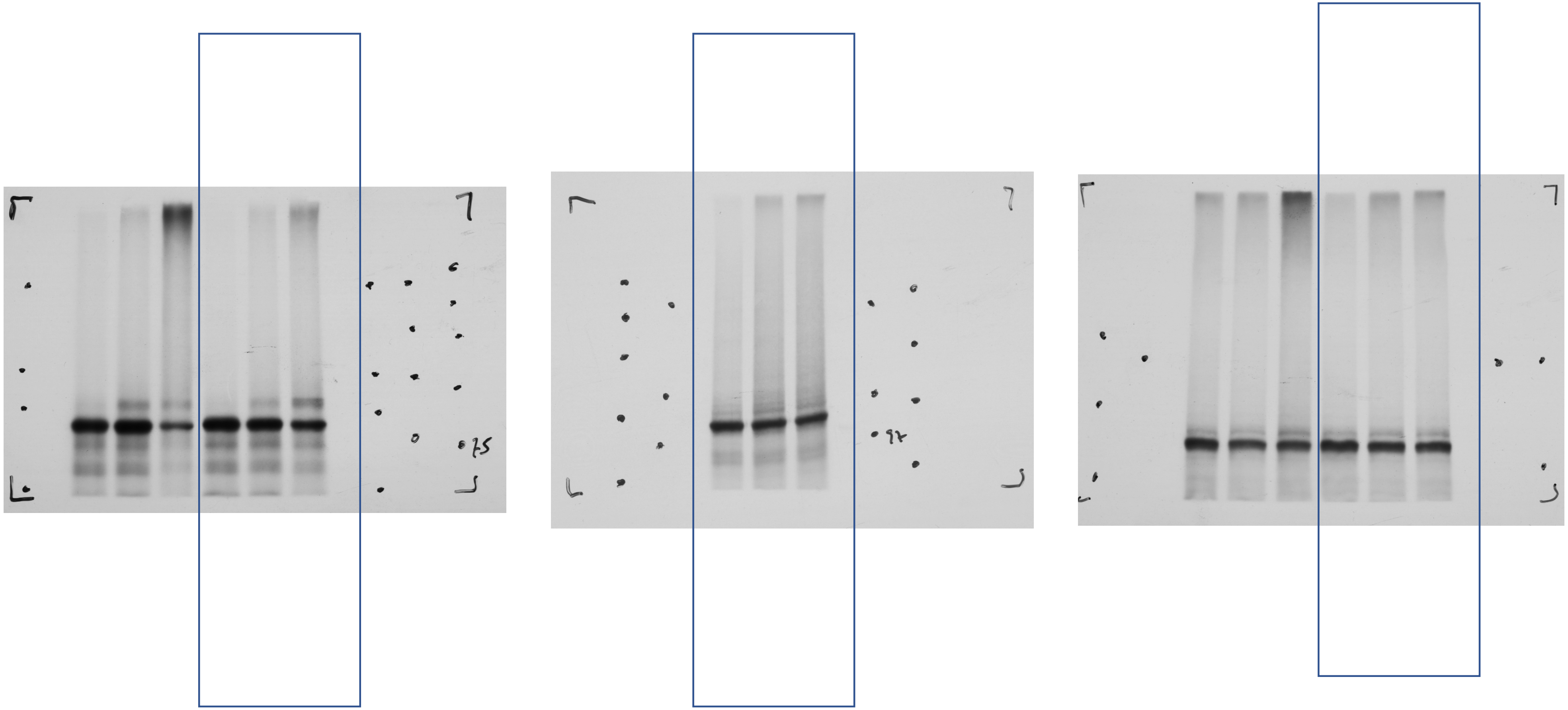

Figure 2C

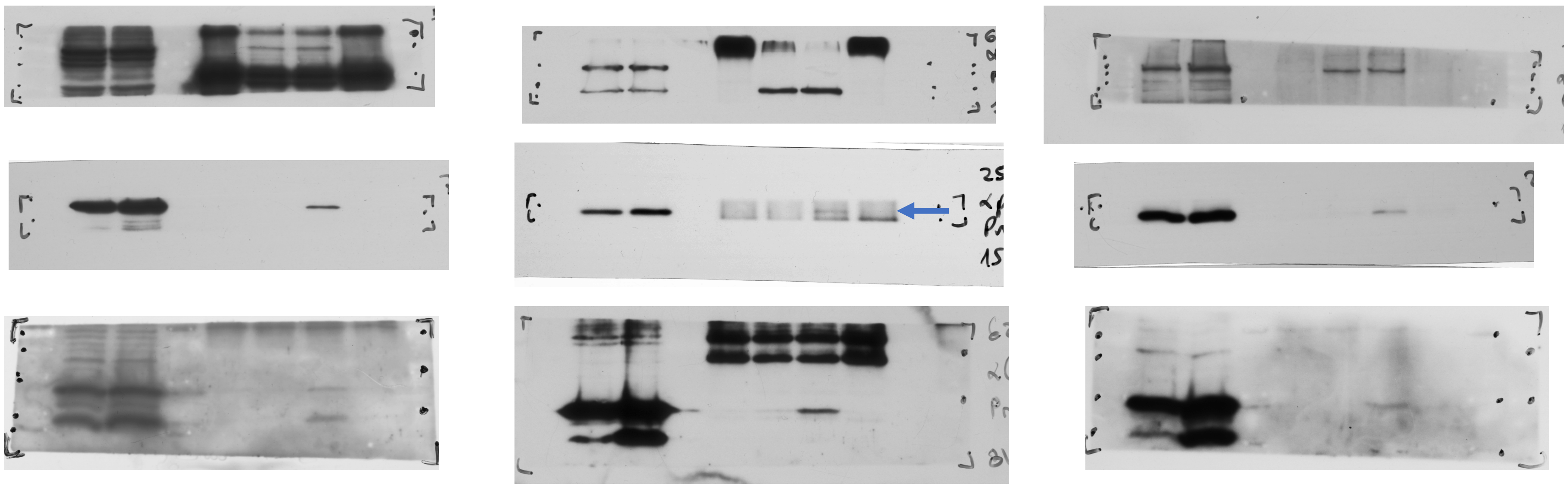

Figure 2D

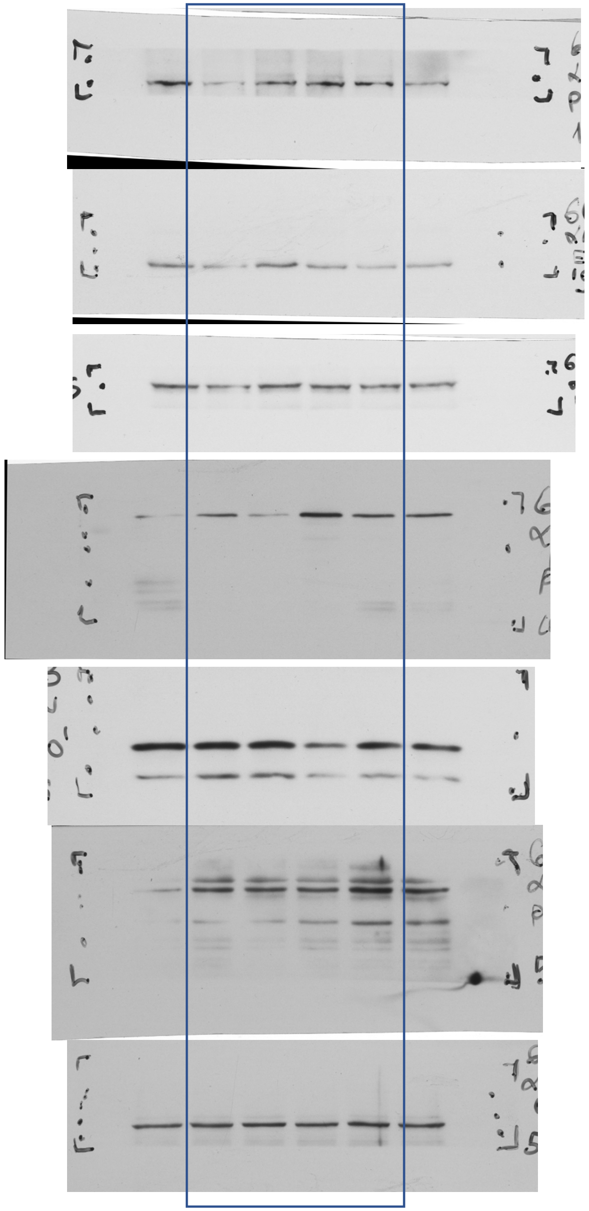

Figure 3A

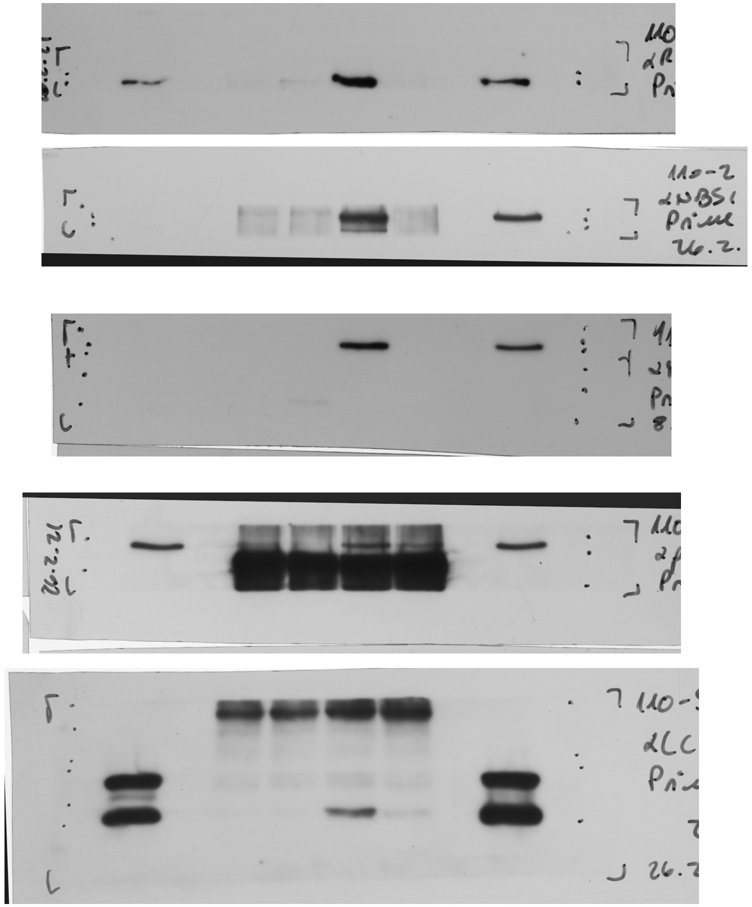

Figure 3C

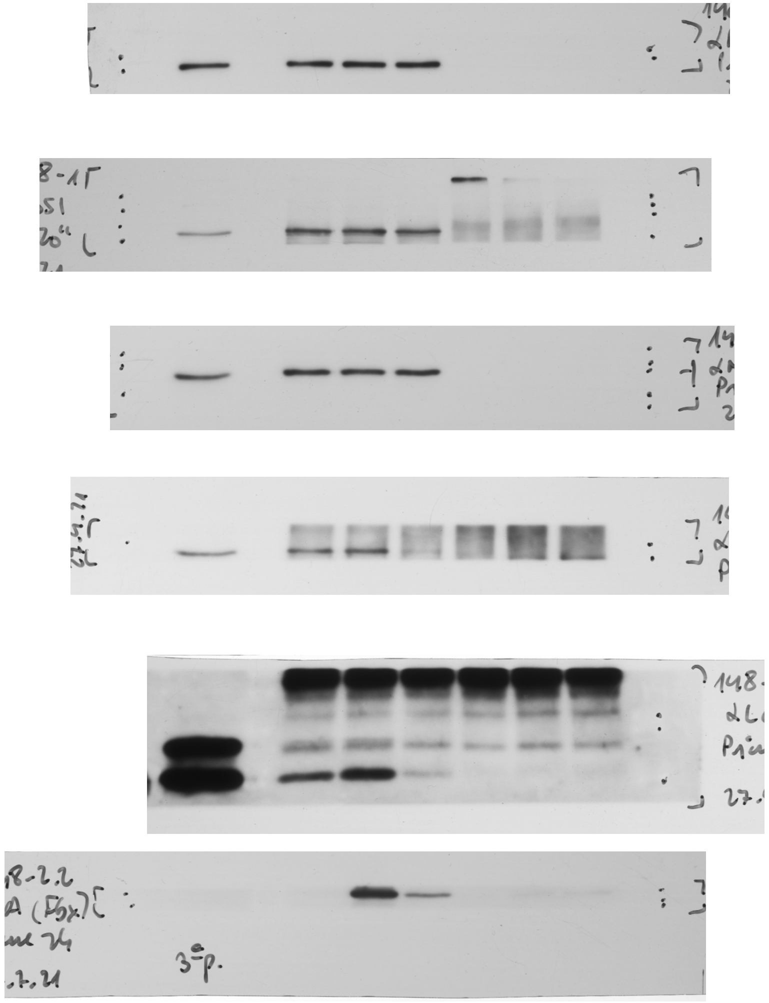

Figure 4A

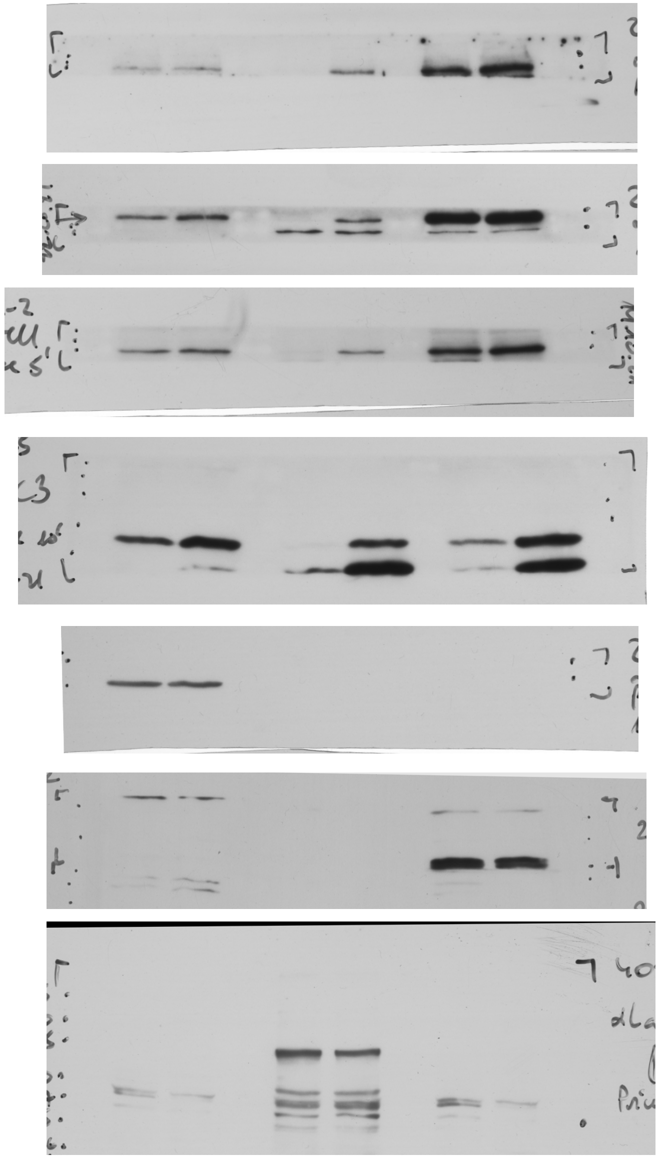

Figure 4B

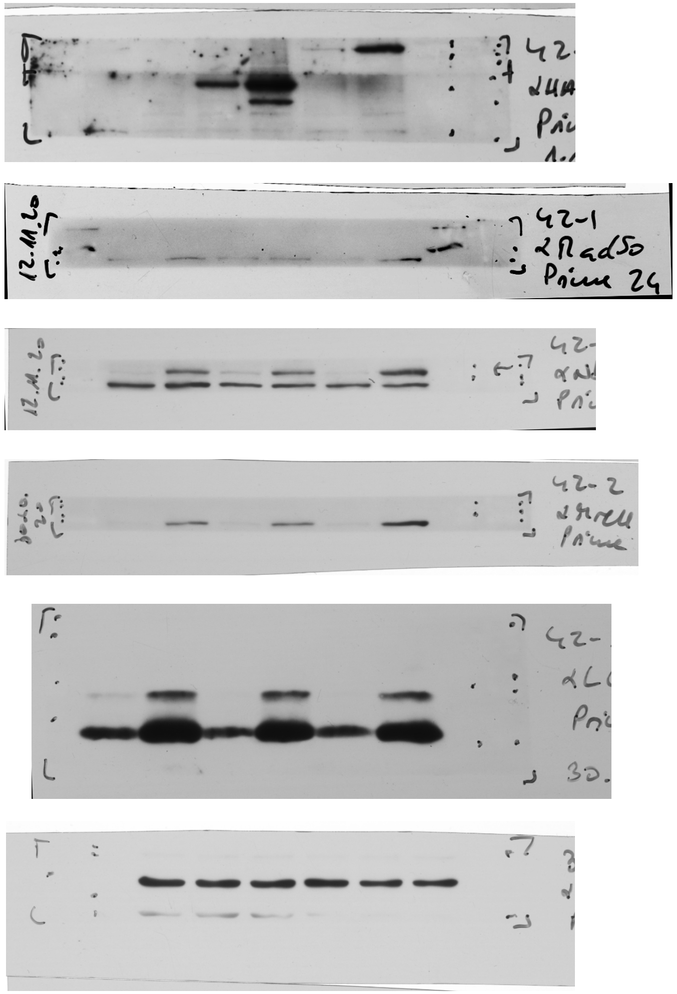

Figure 4C

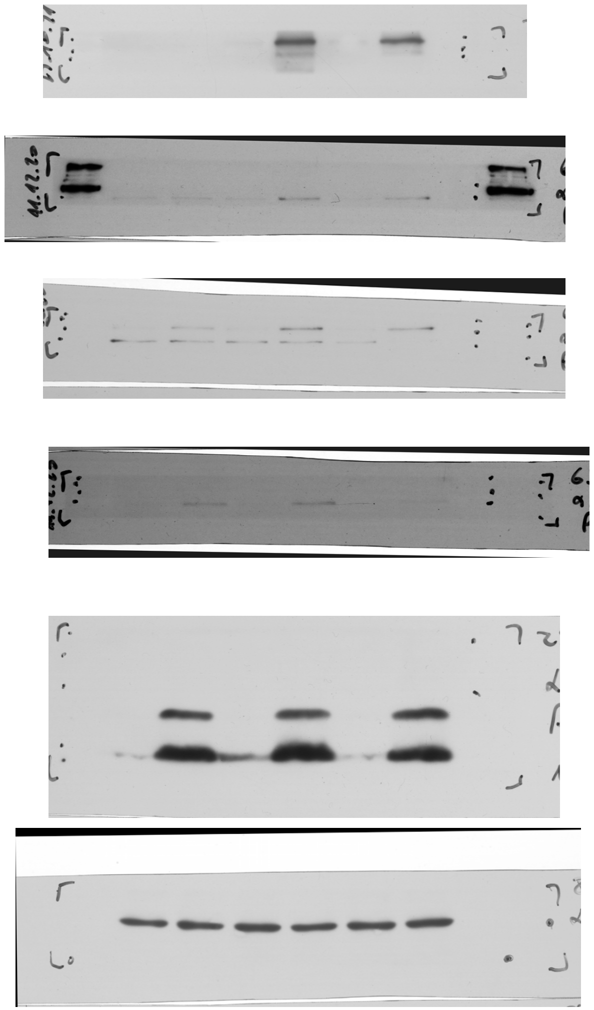

Figure 5A

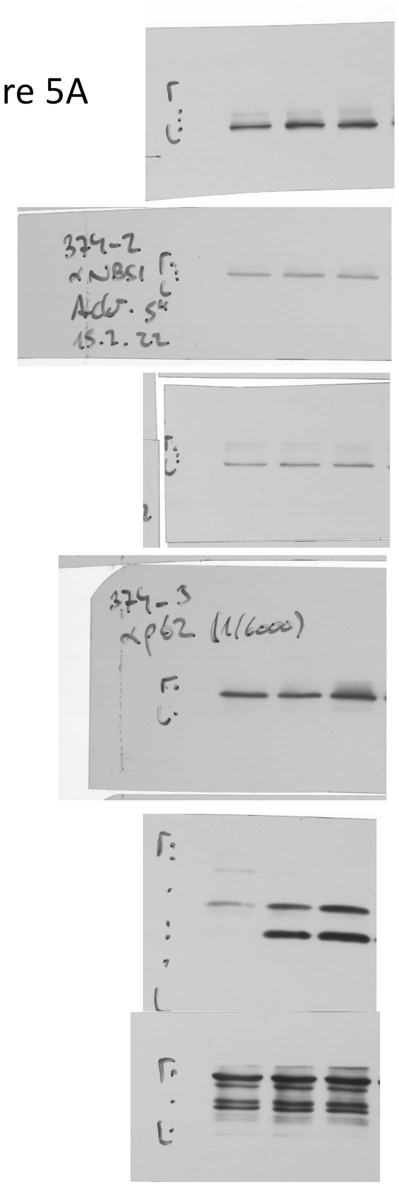

Figure 5B

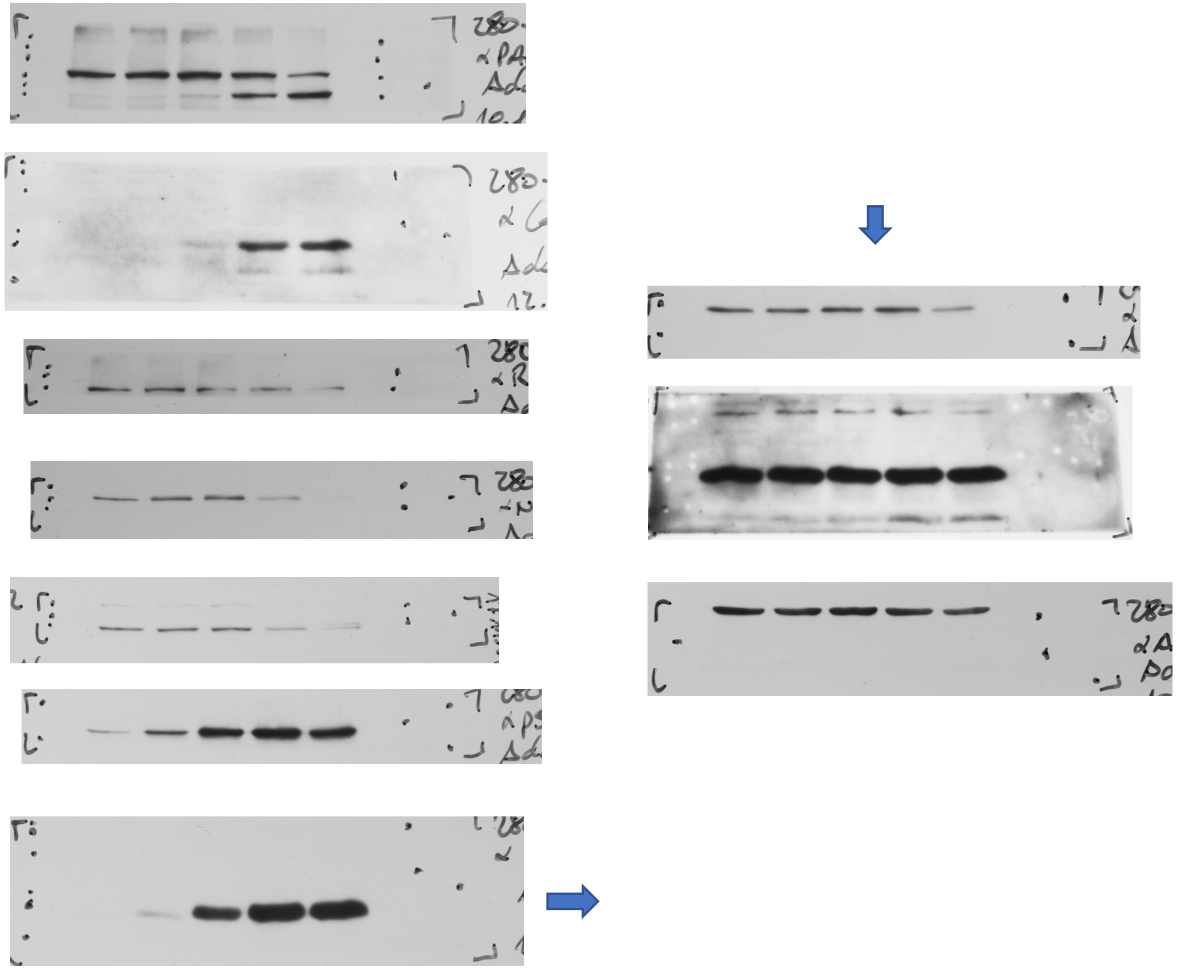

Figure 5D

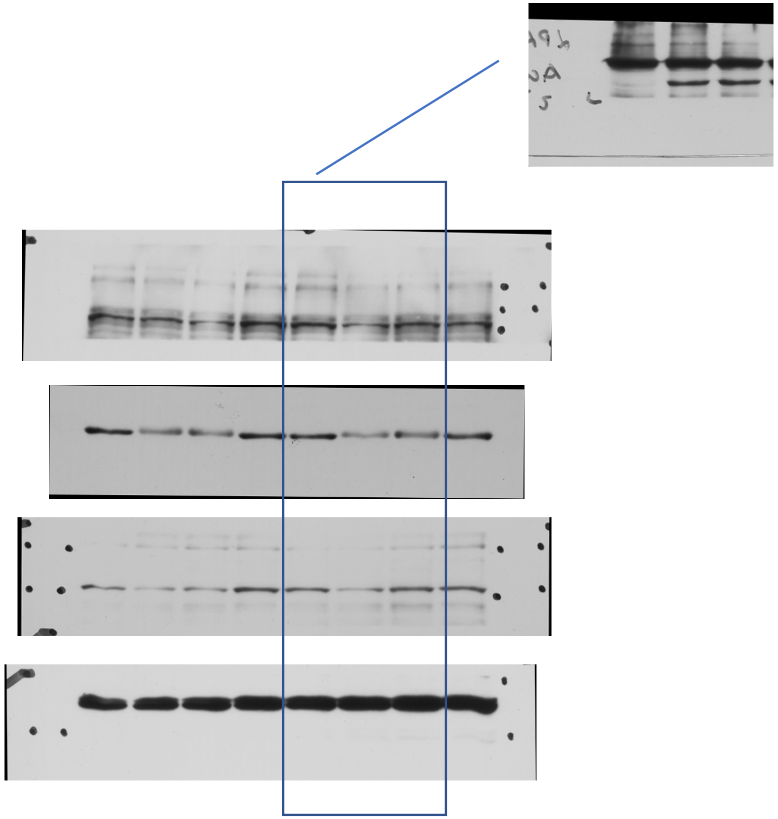

Figure 5E

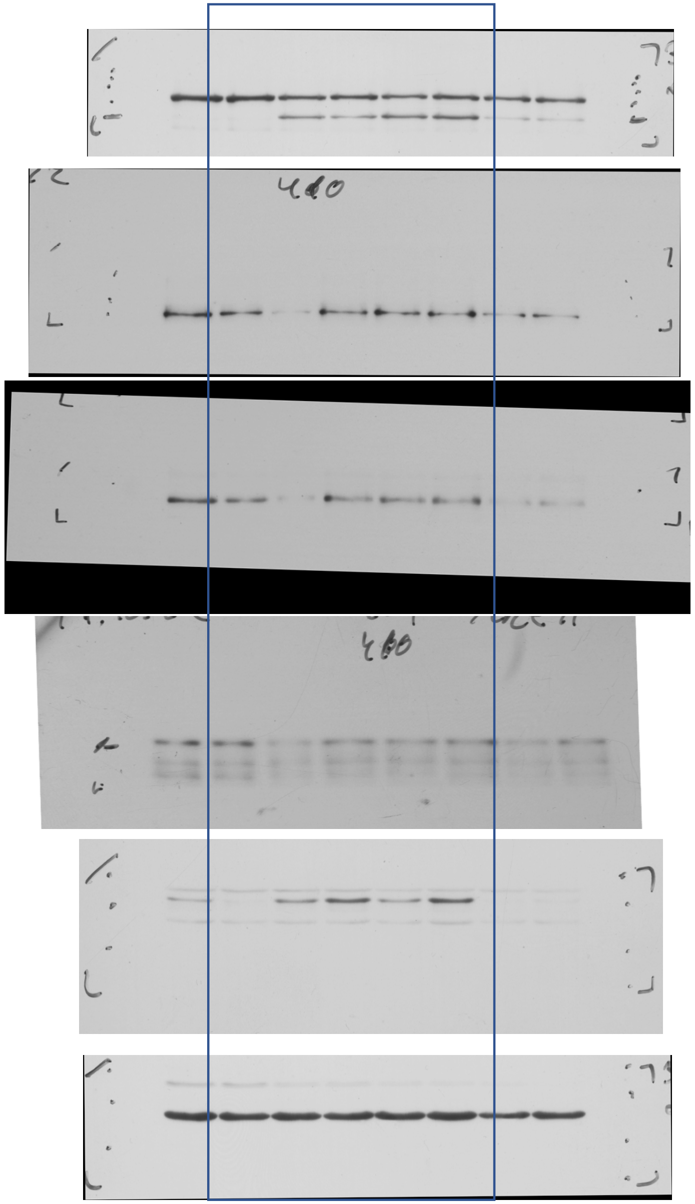

Supplementary Figure S1B

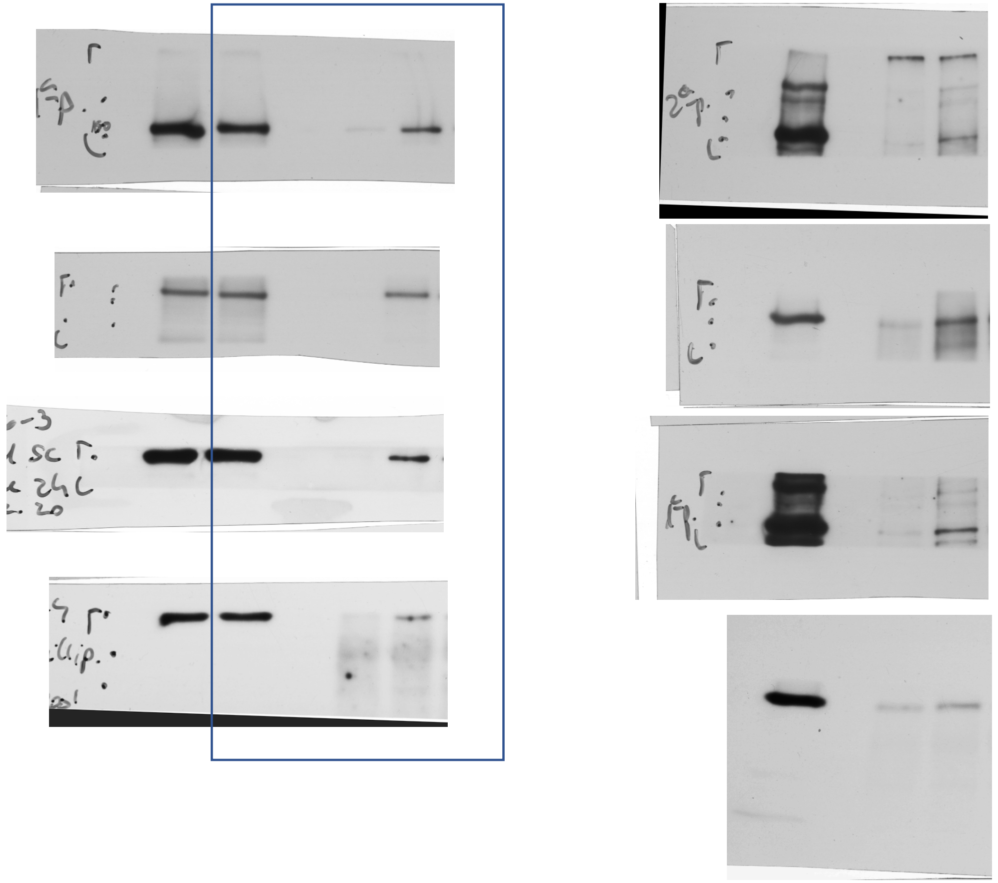

Supplementary Figure S1C

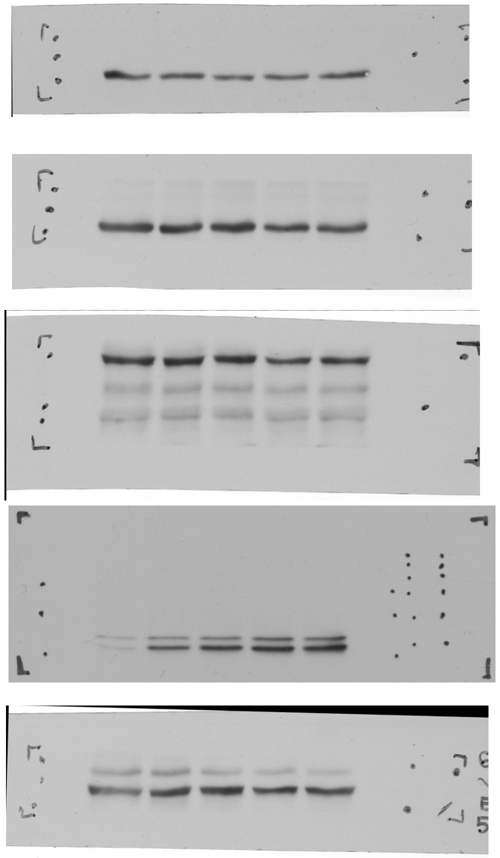

Supplementary Figure S2A

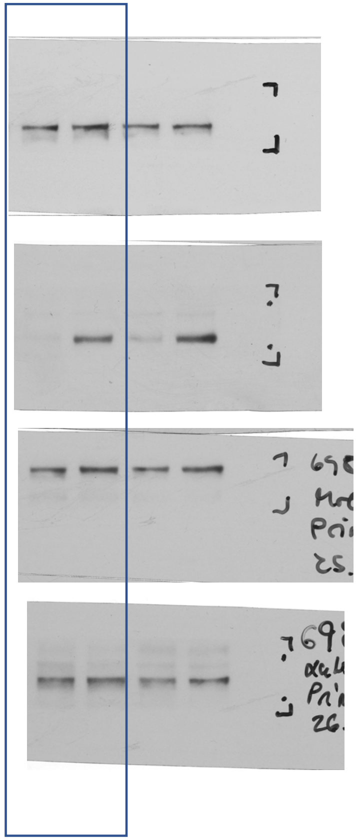

Supplementary Figure S3D

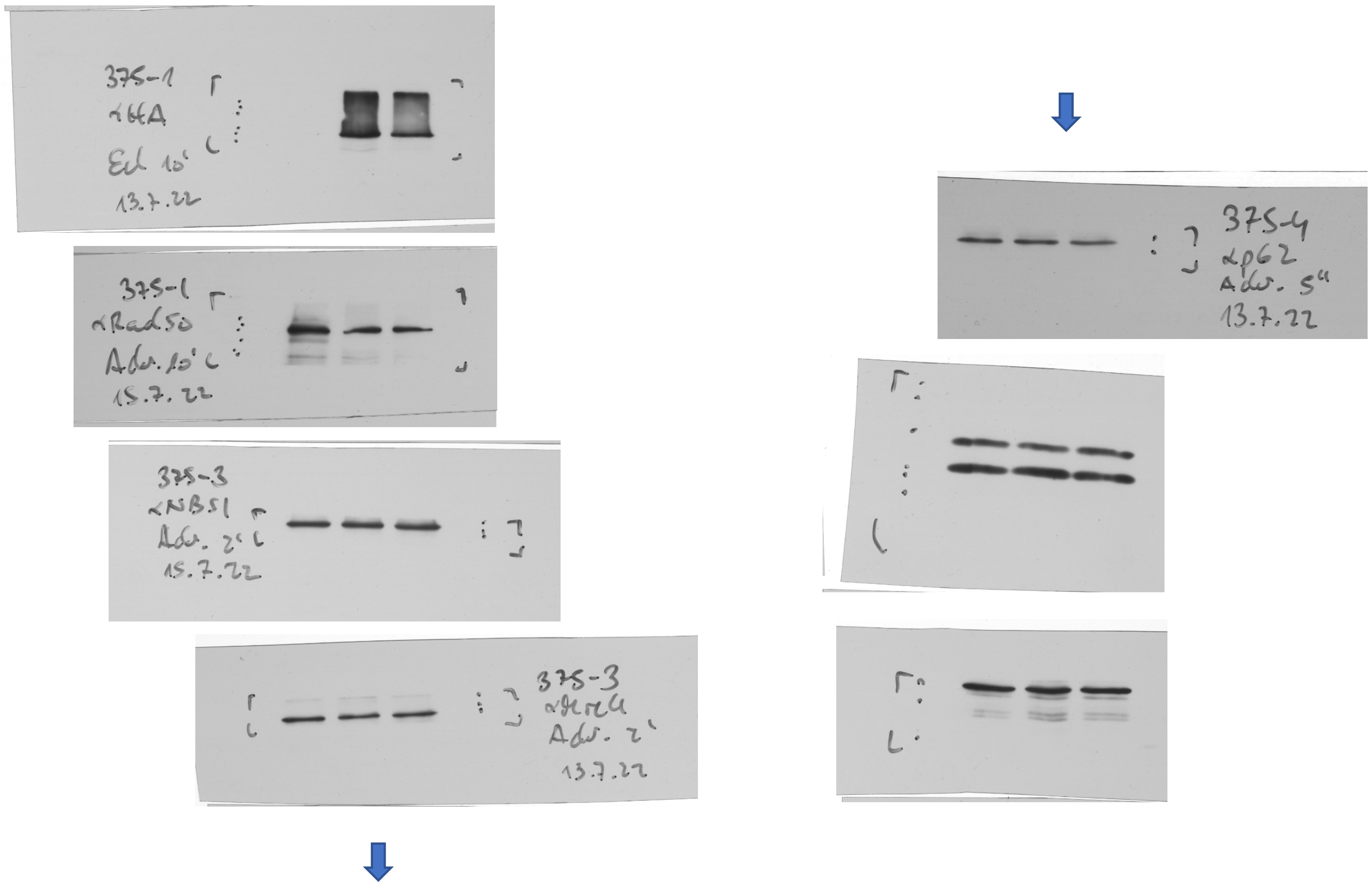

Supplementary Figure S5A

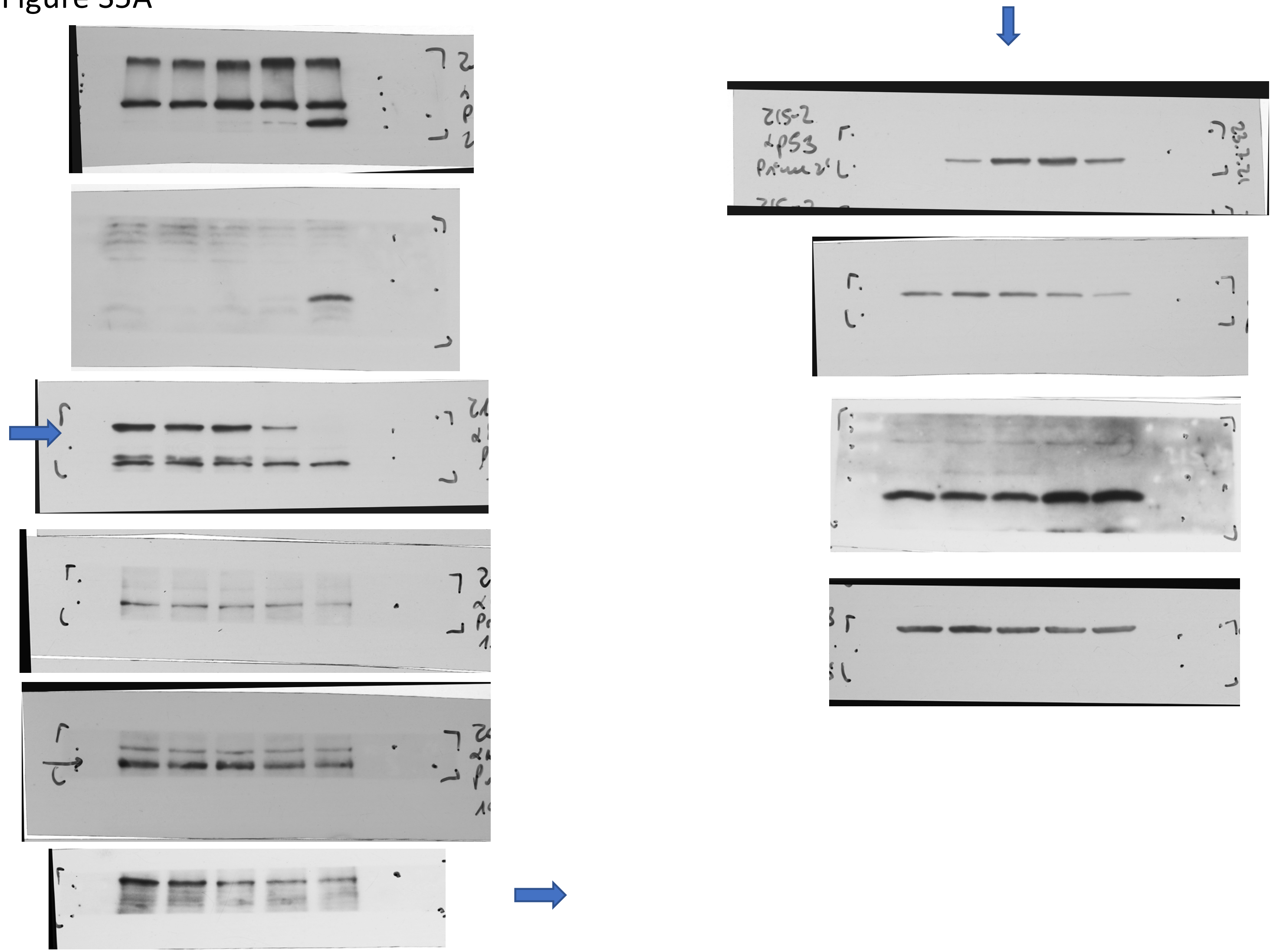

Supplementary Figure S6

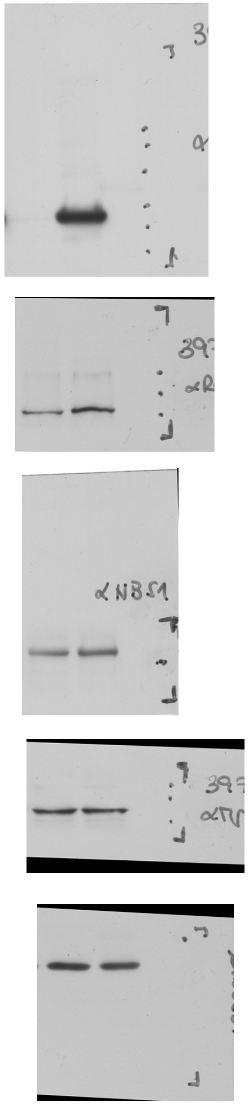

Supplement: Supplementary file 12 — Origunal blots [file 41418_2022_1100_MOESM12_ESM.pdf]
